# Solvent dielectric delimited nitro–nitrito photorearrangement in a perylenediimide derivative†

**DOI:** 10.1039/d2sc02979k

**Published:** 2022-07-04

**Authors:** Aniruddha Mazumder, Ebin Sebastian, Mahesh Hariharan

**Affiliations:** School of Chemistry, Indian Institute of Science Education and Research Thiruvananthapuram Maruthamala P.O., Vithura Thiruvananthapuram Kerala India 695551 mahesh@iisertvm.ac.in

## Abstract

The discovery of vibrant excited-state dynamics and distinctive photochemistry has established nitrated polycyclic aromatic hydrocarbons as an exhilarating class of organic compounds. Herein, we report the atypical photorearrangement of nitro-perylenediimide (NO_2_-PDI) to nitrito-perylenediimide (ONO-PDI), triggered by visible-light excitation and giving rise to linkage isomers in the polar aprotic solvent acetonitrile. ONO-PDI has been isolated and unambiguously characterized using standard spectroscopic, spectrometric, and elemental composition techniques. Although nitritoaromatic compounds are conventionally considered to be crucial intermediates in the photodissociation of nitroaromatics, experimental evidence for this has not been observed heretofore. Ultrafast transient absorption spectroscopy combined with computational investigations revealed the prominence of a conformationally relaxed singlet excited-state (S^CR^_1_) of NO_2_-PDI in the photoisomerization pathway. Theoretical transition state (TS) analysis indicated the presence of a six-membered cyclic TS, which is pivotal in connecting the S^CR^_1_ state to the photoproduct state. This article addresses prevailing knowledge gaps in the field of organic linkage isomers and provides a comprehensive understanding of the unprecedented photoisomerization mechanism operating in the case of NO_2_-PDI.

## Abstract 2

The unprecedented photorearrangement of nitro-perylenediimide (NO_2_-PDI) to nitrito-perylenediimide (ONO-PDI) is shown to occur through a cyclic six-membered transition state triggered by visible-light excitation.

## Graphical abstract

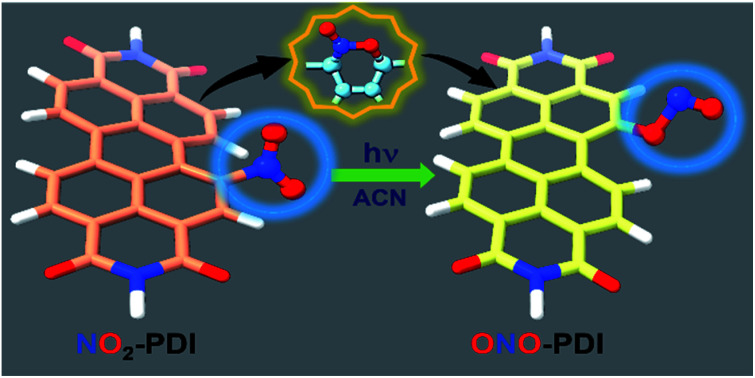

## Introduction

The manifestation of two distinct isomers having the same chemical composition and molecular formula and differing only in the bond connectivity of the ligand to the metal is conventionally termed as linkage isomerism. The intramolecular rearrangement mechanism of nitro–nitrito isomerisation in metal complexes was initially conceptualized by Basolo and Hammaker.^1^ For instance, a nitrite ion (NO_2_^−^) can bind to the metal *via* the N atom as well as through an O atom, giving rise to linkage isomers, which has attracted great attention in the past.^2–7^ Recently, Andrew and co-workers distinguished nitro (–NO_2_) *vs.* nitrito (–ONO) coordination in cytochrome c′, an essential membrane-bound protein in biological systems required for electron transport in the respiratory chain.^8^ Despite the occurrence of nitro–nitrito linkage isomerization being well explored in coordination compounds,^9^ the phenomenon of nitro–nitrito linkage isomerism remains elusive in the case of organic chromophoric systems (selected reports summarized in Table S1†).

The presence of a nitro (–NO_2_) functionality in organic compounds finds multiple potential applications in the form of dyes, explosives, pesticides, polymers, solvents and crucial chemical intermediates.^10^ Rich excited-state photophysics, along with diverse photochemistry, makes nitroaromatics (NAs) one of the most riveting classes of compounds among the vast plethora of organic molecular architectures. Predominantly, NAs are reported to be non-fluorescent due to the following major excited-state processes: (i) population of triplet manifolds (T_n_) *via* ultrafast intersystem crossing (ISC); (ii) ultrafast non-radiative deactivation to the ground-state (S_0_) and (iii) photochemical transformations of the NA compounds.^11–14^ Among these, the most intriguing aspect of NAs is photochemical dissociation/degradation, as nitro compounds constitute major environmental pollutants, leading to adverse climatic changes as well as fatal DNA mutations in cellular systems.^15–18^ Biodegradation of even the simplest NA compounds, such as nitro-benzene and *ortho*-nitrophenol, is strenuous and poses severe threats to marine living organisms.^19–21^ That being said, scientists in the past have extensively studied the divergent photochemistry of NAs with associated excited-state dynamics in the solution state. Most commonly, the photochemistry of NAs is governed by the dissociation of the molecule to nitrosyl (NO^˙^) and aryloxy (ArO^˙^) free radicals, which may combine bimolecularly to give multiple secondary photoproducts.^22^ It is rationalized that the photodissociation step is probably characterized by a nitritoaromatic intermediate (–ONO), whose experimental characterization is still under debate and has not been hitherto observed.^11^

**Fig. 1:**
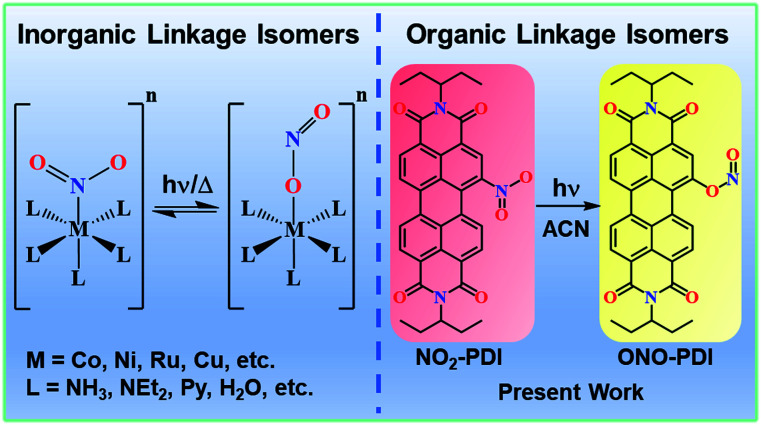
Schematic representations of inorganic (left) and organic (right) linkage isomers.

Our persistent efforts to understand novel non-covalent interactions in unique chromophoric architectures^23,24^ and decipher the diverse photoexcited state processes in multi-chromophoric assemblies^25–28^ motivated us to consider the realm of linkage isomers and thereby unravel the photoexcited mechanism responsible for the isomerization. This report highlights the first unequivocal evidence of linkage isomerism in a nitro-perylenediimide chromophore. The visible-light-driven photorearrangement of nitro-perylenediimide (NO_2_-PDI) to nitrito-perylenediimide (ONO-PDI) is studied in a polar aprotic acetonitrile solvent (ACN, Fig. 1). ONO-PDI was isolated and thoroughly characterized using standard spectroscopic, spectrometric and elemental composition techniques. The stability of the nitrito isomer was found to be lower compared to that of the nitro isomer, and this result is in excellent agreement with the previously reported stabilities of nitrite linkage isomers.^29^ The numerous possibilities of NA photochemistry in solvents with varying dielectric properties were well established by Mathew, Prakash and co-workers,^30^ supporting the solvent dependency of photoisomerization observed in NO_2_-PDI. The never-ending interest in the excited-state properties of NAs mainly stems from the ultrafast dynamic branching of the singlet excited Franck–Condon state to either the triplet receiver or photoproduct states. Furthermore, even the smallest and simplest nitrobenzene exhibits very complex photophysics, making it hard to decipher the photochemistry originating from the photoexcited states.^31,32^ Our incessant efforts towards interpreting the underlying mechanism responsible for the photoisomerization of NO_2_-PDI impelled us to perform an in-depth analysis of the femtosecond (fsTA) and nanosecond (nsTA) transient absorption spectroscopic measurements of NO_2_-PDI in solution. The results obtained from the fsTA and nsTA experiments corroborated the role played by a conformationally relaxed singlet excited-state in the photoisomerization mechanism.^33–35^ According to Chapman's orientation–photoreactivity formulations, the nitro-aromatic torsional angle in the relaxed singlet excited-state either favours or disfavours the orbital interactions essential to the photochemical transformations.^36^ Considering Chapman's criteria, our theoretical calculations show the possibility of a sterically stable six-membered cyclic transition state acting as an intermediate between the relaxed singlet excited-state and the photoproduct state. There exists a strong correlation between the dielectric properties of the solvent and the stability of the proposed transition state. This is exemplified by the high rate of photoisomerization of NO_2_-PDI in acetonitrile, followed by the non-observance of photoisomerization in non-polar toluene.

## Results and discussion

### Synthesis and characterization

**Fig. 2:**
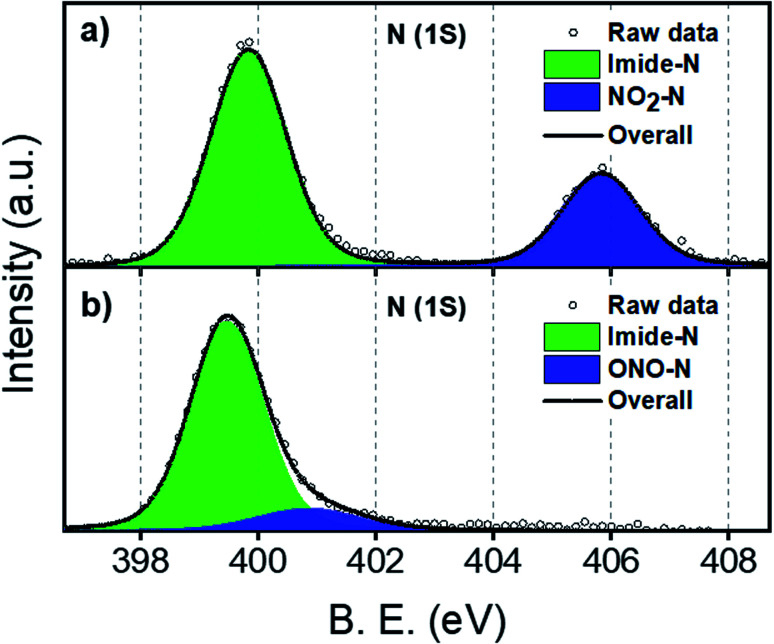
Core-level N (1s) XPS spectra for (a) NO_2_-PDI and (b) ONO-PDI in the powder state.

NO_2_-PDI was synthesized and characterized as per a previous report in the literature.^37^ONO-PDI was synthesized by irradiating NO_2_-PDI with a 532 nm, 10 mW diode laser (2 h) in the solvent ACN, followed by silica gel column purification (EtOAc as eluent), affording a yellow-coloured solid (Fig. S1†). X-ray photoelectron spectroscopy (XPS) was used to elucidate the different bonding patterns and the chemical states of the bonded elements in the linkage isomers NO_2_-PDI and ONO-PDI. Both the isomers exhibited significant changes in the binding energy value of the N (1s) XPS peaks. The XPS N (1s) core level spectrum of powder samples of NO_2_-PDI and ONO-PDI were recorded using a Mg Kα X-ray source with a power of 100 W. Deconvolution of the N (1s) XPS spectrum of NO_2_-PDI (Fig. 2a) reveals the existence of two distinct peaks with binding energy values, *E*_b_ = 399.85 eV and *E*_b_ = 405.85 eV. These two peaks establish the presence of two nitrogen atoms in different chemical states in NO_2_-PDI. The peak possessing a lower binding energy of *E*_b_ = 399.85 eV corresponds the imide-nitrogen atom, whereas the higher binding energy peak at 405.85 eV unambiguously proves the different chemical state of the nitrogen atom in the nitro group, which correlates well with the previous report in the literature.^38^ On the other hand, the N (1s) XPS spectrum of ONO-PDI (Fig. 2b) exhibits two prominent peaks at binding energy values of *E*_b_ = 399.45 eV and *E*_b_ = 400.85 eV. The N (1s) peak at 399.45 eV corresponds to the imide-nitrogen atom, whose chemical state is unchanged in both the isomers. The second peak at 400.85 eV confirms the presence of the nitrogen atom in the nitrito group having a different chemical state with respect to the nitrogen atom of the nitro group in the NO_2_-PDI isomer.^39^ The momentous shift (Δ*E*_b_ = 5 eV) observed in the binding energy value of the second N (1s) peak going from ONO-PDI to NO_2_-PDI strongly corroborates the structural characterization of the isomers, and could possibly be the manifestation of the nitrogen atom attaining an sp-hybridized state in NO_2_-PDI.^40^ Detailed structural examination of the isomers based on the C (1s) and O (1s) core level XPS analysis can be found in the ESI (Fig. S2†).

**Fig. 3:**
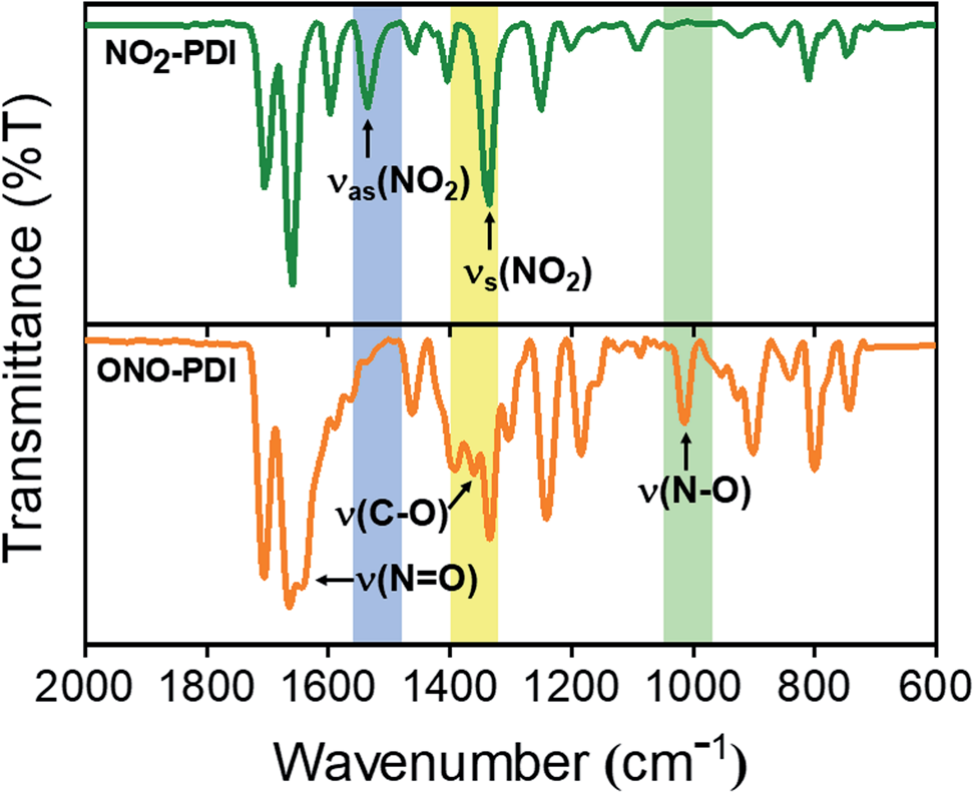
FTIR spectra of NO_2_-PDI (top) and ONO-PDI (bottom) in KBr disks.

Fourier-transform infrared spectroscopy (FTIR) was performed to investigate the vibrational signatures in NO_2_-PDI and ONO-PDI (Fig. 3). The IR spectrum of NO_2_-PDI reveals the characteristic asymmetric and symmetric stretching frequencies of the –NO_2_ group at *ν* = 1535 cm^−1^ and *ν* = 1342 cm^−1^, respectively. The IR spectrum of ONO-PDI displayed unique vibrational features at *ν* = 1015 cm^−1^, *ν* = 1360 cm^−1^ and *ν* = 1643 cm^−1^. The presence and absence of the vibrational peak at *ν* = 1015 cm^−1^ in ONO-PDI and NO_2_-PDI, respectively, confirms the N–O bond stretching characteristic of the nitrito (ONO) group.^41^ The C–O stretching peak at *ν* = 1360 cm^−1^ and the N=O stretching peak at *ν* = 1643 cm^−1^ in the IR spectrum of ONO-PDI further confirm the structural characterization of the linkage isomers. Evidence for the transformation of the –NO_2_ functionality to an –ONO functionality in the photorearrangement of nitro-perylenediimide was examined using Raman spectroscopy. Data acquisition was carried out on powder samples of NO_2_-PDI and ONO-PDI with photoexcitation at 632.8 nm and an acquisition time of 5 s using a 50× objective. The intense peak centred at ∼1371 cm^−1^ in the Raman spectrum of NO_2_-PDI (Fig. S3a†) reveals the presence of a vibrational band corresponding to the –NO_2_ symmetric stretch. The observance of the Raman peak centred at ∼1343 cm^−1^ and the absence of the intense ∼1371 cm^−1^ peak in the Raman spectrum of ONO-PDI (Fig. S3b†) confirm the structural difference between the two isomers.^42^ The Raman peak at 1343 cm^−1^ is assigned to the symmetric stretching vibration of the –ONO group. The large shift (∼28 cm^−1^) observed in the Raman peaks going from the –NO_2_ symmetric stretch to the –ONO symmetric stretch mainly originates from the very highly electron-withdrawing nature of the nitro group compared to that of the nitrito group.^43^ Detailed structural characterization of both the isomers using standard nuclear magnetic resonance (^1^H-NMR), CHN elemental microanalysis and high resolution mass spectrometry (HRMS) methods are provided in the ESI (Fig. S4–S6, Table S2 and Fig. S7, respectively).†

### Optical properties

Steady-state absorption spectroscopy was used to explore the ground-state electronic properties of NO_2_-PDI and ONO-PDI in chloroform (*C*_0_ ≅ 0.5–1 μM) at room temperature. The UV-vis absorption spectrum of NO_2_-PDI shows the characteristic spectral signatures of the parent perylenediimide (PDI) chromophore, with a 4 nm hypsochromic shift in the absorption maximum (Fig. S8a†). The S_0_–S_1_ electronic transition (*f* = 0.7612, Table S3 and Fig. S9a†) in NO_2_-PDI was characterized by the absorption maxima at *λ*^Abs^_max_ ≅ 521 nm. The broadening of the higher-energy vibronic bands in the absorption spectrum of NO_2_-PDI at *λ*_0–_^Abs^_1_ ≅ 489 nm and *λ*_0–_^Abs^_2_ ≅ 454 nm possibly arises from the strong vibronic coupling induced by the nitro (–NO_2_) group to the perylene core.^11,44^ The resonance Raman spectrum of NO_2_-PDI under 488 nm excitation displayed the presence of a strong –NO_2_ symmetric stretching vibrational mode at 1371 cm^−1^ (Fig. S10†), corroborating the effect of vibronic coupling responsible for the broadening of the higher-energy vibronic bands in NO_2_-PDI absorption spectrum.^45^ In validation with the theoretical predictions, the UV-vis absorption spectrum of ONO-PDI reveals highly blue-shifted absorption peaks in comparison to those in the spectrum of NO_2_-PDI. The S_0_–S_1_ electronic transition (*f* = 0.8073, Table S3 and Fig. S9b†) in ONO-PDI showed an absorption maximum at *λ*^Abs^_max_ ≅ 377 nm. The other vibronic peaks were observed at *λ*^Abs^_0–1_ ≅ 356 nm and *λ*^Abs^_0–2_ ≅ 346 nm. The broad red-shifted peak having a maximum at ≅416 nm in the absorption spectrum of ONO-PDI could possibly be due to a weak charge-transfer (CT) transition as a result of electron delocalization from the lone pairs of the oxygen atom in the –ONO moiety to the electron-deficient perylene core.^46^ Steady-state fluorescence spectroscopy was performed to understand the photoluminescence properties of the linkage isomers NO_2_-PDI and ONO-PDI (*C*_0_ ≅ 0.5–1 μM) at room temperature in chloroform (Table S4†). NO_2_-PDI was found to be very weakly emissive with a fluorescence quantum yield (*Φ*_F_) of less than 1% as compared to parent PDI chromophore and an emission maximum at *λ*^Em^_max_ ≅ 458 nm (Fig. S8b–c†). The strong vibronic coupling induced by the nitro (–NO_2_) group to the perylene core together with the nitro-aromatic torsion angle relaxation in the excited-state could be associated with the quenching of fluorescence in NO_2_-PDI (the emission spectrum could only be obtained using a higher slit width of 1.5 mm and a high concentration of the sample). Likewise, ONO-PDI showed weak fluorescence characteristics with a fluorescence quantum yield (*Φ*_F_) of less than 1% and an emission maximum at *λ*^Em^_max_ ≅ 459 nm (Fig. S9b†). The possibility of a weak CT transition was also observed based on the broad red-shifted emission peak in the emission spectrum of ONO-PDI, which had a maximum at ∼588 nm.

### Photoirradiation experiments

**Fig. 4:**
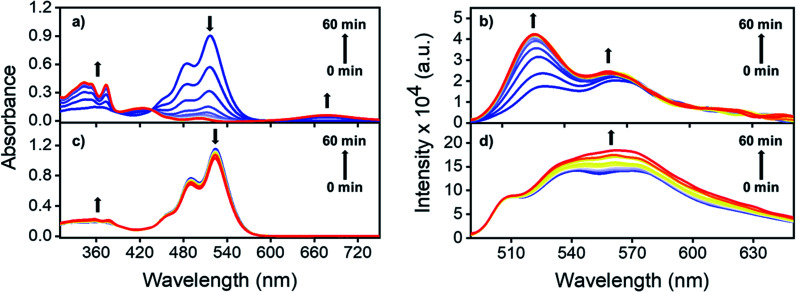
Time-dependent laser-irradiated UV-vis absorption spectra of NO_2_-PDI in (a) acetonitrile and (c) toluene. Time-dependent laser irradiated emission spectra (exc. at 532 nm) of NO_2_-PDI in (b) acetonitrile and (d) toluene.

To understand the role played by the dielectric properties of the solvent in photoisomerization, time-dependent (TD) laser irradiation studies were carried out on NO_2_-PDI in ACN and toluene (TOL, Fig. 4).^47^ The photoisomerization process was monitored using the UV-vis absorption and photoluminescence techniques upon irradiation with a 532 nm, 10 mW diode laser. Laser irradiation studies of NO_2_-PDI in ACN (*ε* = 37.5) revealed the rapid photochemical transformation of NO_2_-PDI, which was observed as the decrease in the intensity of the *λ*^Abs^_max_ (521 nm) peak and the gradual increase in the intensity of the *λ*^Abs^_max_ (350 nm) peak, as reflected in the absorption spectrum over a total span of one hour (Fig. 4a). The evolution of the red-shifted broad band in the TD NO_2_-PDI absorption spectrum indicates the formation of a PDI radical anion (NO_2_-PDI)^˙−^.^48^ The TD emission spectrum in ACN (Fig. 4b) displays a significant enhancement of fluorescence intensity, along with the progression of well-structured emission bands. On the contrary, the TD studies in TOL (*ε* = 2.4) portray a completely different situation. NO_2_-PDI exhibited excellent photochemical stability in TOL under the same experimental conditions as ACN. The TD UV-vis absorption spectrum (Fig. 4c) showed almost negligible transformation of NO_2_-PDI to ONO-PDI in non-polar TOL. Additionally, minute enhancement in the fluorescence intensity was observed in the TD emission spectrum of NO_2_-PDI in TOL (Fig. 4d), which suggested very low yields of photoisomerization. The feasibility of the isomerization process solely *via* thermal energy in the absence of light was investigated using controlled heating experiments.^49–51^ Two NO_2_-PDI solutions of the same concentration were prepared in ACN and TOL, and were completely covered with aluminium foil to avoid any unwanted photoexcitation. Both the solutions were heated at 50 °C for 30 min, followed by heating at 70 °C for 30 min and finally heating at 150 °C for 10 min. No visible changes were discerned in the colour of the solutions; additionally, the UV-vis absorption spectra after heating did not display any characteristic signatures of ONO-PDI in either of the solutions (Fig. S11†). This study certified that the observed isomerization of NO_2_-PDI is not achievable in the presence of thermal energy alone, even at high temperatures.

### Femtosecond transient absorption (fsTA) studies

**Fig. 5:**
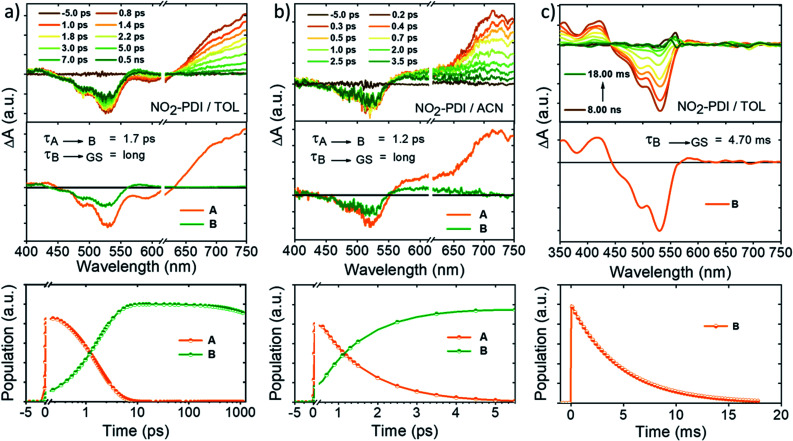
(top) fsTA spectra (*λ*_ex_ = 532 nm) of NO_2_-PDI in (a) TOL and (b) ACN showing the excited-state dynamics after photoexcitation at 532 nm. (middle) Evolution associated spectra reconstructed from global analysis of the time *vs.* wavelength based three-dimensional fsTA spectrograph. (bottom) Relative population profiles of the excited states fitted using kinetic models. (c) (top) nsTA spectra (*λ*_ex_ = 532 nm) of NO_2_-PDI in TOL. (middle) Evolution associated spectra reconstructed from global analysis of the time *vs.* wavelength based three-dimensional nsTA spectrograph. (bottom) Relative population profile of the excited-state (B) fitted using kinetic models.

Our continuous efforts to understand the multifarious behaviour of the excited-state dynamics in different dielectric environments propelled us to probe the nature and fate of the excited states in NO_2_-PDI after photoexcitation by performing fsTA measurements in the solvents TOL and ACN (optical density = 0.2–0.3, photoexcited at 532 nm with a 100 fs laser pulse). The kinetic components responsible for these transformations in the NO_2_-PDI fsTA spectra were extracted through a singular value decomposition (SVD) method followed by global analysis of the time *vs.* wavelength based three-dimensional map of the fsTA spectra.^52^ In global analysis, the entire wavelength range was analysed synchronously, and a sequential model was employed to obtain evolution associated spectra (EAS). Upon photoexcitation, the fsTA spectra of NO_2_-PDI in nitrogen-purged TOL (Fig. 5a-top) exhibited the characteristic transient features of the parent PDI chromophore. Negative ground-state bleach (GSB) and weak stimulated emission (SE) were observed in the fsTA spectra at ∼450 nm to 614 nm.^53,54^ A positive broad excited-state absorption (ESA) band was found at ∼615 nm to 750 nm, which decays on an ultrafast timescale to give rise to a new transient species. The newly evolved transient species is characterized by positive ESA in the visible region from ∼560 nm to 615 nm. NO_2_-PDI in TOL displayed two principal components (Fig. 5a-middle); the first component (A) is ascribed to the locally excited singlet state (S_1_–S_*n*_ electronic transitions), which decays within 1.7 ± 0.3 ps to give rise to the second principal component (B). Component (B) was found to be a long-lived species whose decay was not complete within the fsTA experimental time window. Similarly, fsTA measurements of NO_2_-PDI in ACN exhibited GSB at ∼450 nm to 552 nm and a positive broad ESA band at ∼551 nm to 750 nm (Fig. 5b-top). The broad ESA band decayed on an ultrafast timescale, giving rise to a new transient species. The newly formed excited-state species is characterized by broad positive absorption at ∼550 nm to 660 nm. NO_2_-PDI in ACN featured two principal components (Fig. 5b-middle). The first component (A) is assigned to the locally excited singlet state (S_1_–S_*n*_ electronic transitions), and decays with a time constant of 1.2 ± 0.2 ps, giving rise to the second principal component (B), which exhibits a long lifetime analogous to the long-lived component observed in the fsTA measurement of NO_2_-PDI in toluene.

### Nanosecond transient absorption (nsTA) studies

To shed light on the nature of the long-lived species (B) observed in the fsTA experiment of NO_2_-PDI, nanosecond-transient absorption (nsTA) studies were executed in both the non-polar solvent TOL (*ε* = 2.4) and the polar aprotic solvent ACN (*ε* = 37.5). Upon photoexcitation at 532 nm with an 8–10 ns laser pulse, the nsTA spectra of NO_2_-PDI in nitrogen-purged TOL displayed the presence of strong positive ESA bands at ∼360 nm and ∼420 nm (Fig. 5c-top). Along with this, intense GSB was observed at ∼440 nm to 565 nm accompanied by a weak positive ESA band at ∼565 nm to 610 nm. On the other hand, the nsTA spectra of NO_2_-PDI in nitrogen-purged ACN showed similar spectral features to those observed in TOL, consisting of weak ESA bands at ∼356 nm and ∼410 nm (Fig. S12†). In addition to ESA, strong GSB was also observed in the nsTA spectra of NO_2_-PDI in ACN ranging from ∼440 nm to 570 nm (the post-experiment UV-vis absorption spectrum of NO_2_-PDI showed that a small fraction of the sample had undergone photoisomerization). To further probe into the nature of the ESA band, we performed nsTA measurements in oxygen-purged toluene solutions (Fig. S13†).^55^ To our surprise, quenching of the ESA intensity was not observed even after long purging with oxygen gas. Further, triplet–triplet energy transfer analysis of NO_2_-PDI with various triplet quenchers having a lowest triplet state energy higher and lower than that of the lowest triplet state of NO_2_-PDI (1.2 eV) rules out the possibility of the population of a triplet excited-state of NO_2_-PDI in TOL solution upon photoexcitation (Fig. S14–S16 and Table S5†).^56^ To obtain deeper insights into the theoretical possibility of populating a triplet excited-state of NO_2_-PDI, spin–orbit coupling (SOC) values were calculated using the PySOC package implemented in Gaussian 16, computed at the CAM-B3LYP/6-311++G(d,p) level of theory. The SOC values were found to be small and insignificant, rationalizing the infeasibility of triplet formation in the NO_2_-PDI molecule (Table S6†).^57^ Previously, it has been reported by Vauthey and co-workers that moving from smaller NAs like nitro-naphthalene and nitro-anthracene to larger NAs like nitro-perylene, the chances of populating the triplet state *via* ISC are very minute and therefore, other deactivation channels from the singlet excited-state to the ground state become more favourable.^12^

Identification of the kinetic components corresponding to the photoprocesses observed in the nsTA spectra of NO_2_-PDI was conducted using the SVD method, followed by global analysis of the time *vs.* wavelength based three-dimensional map of the nsTA spectra. The EAS of NO_2_-PDI exhibited a single principal component (B) in TOL (Fig. 5c-middle; global analysis of the nsTA spectra in ACN was not performed due to the photoisomerization/degradation of the NO_2_-PDI sample during the measurement). Fitting of the single component (B) yielded an unusually long decay lifetime of 4.70 ± 0.01 ms in TOL. The persistence of the long-lived transient feature of NO_2_-PDI in TOL and ACN over the temperature range from room temperature to −10 °C rules out the possibility that the long-lived transient species is a photochemical species (Fig. S17 and S18†). To have a better understanding of the character of the long-lived species obtained in the nsTA experiment of NO_2_-PDI, we proceeded to investigate the possibility of a radical anion (NO_2_-PDI)^˙−^ excited-state of nitro-perylenediimide, which could be the reason for the ESA band in the nsTA experiment. The feasibility of forming a stable radical anion of NO_2_-PDI in highly polar solvents like dimethylformamide (DMF) was already showcased by Apurba and co-workers in 2018.^48^ With this motivation, we carried out the chemical reduction of NO_2_-PDI using cobaltocene (CoCp_2_) followed by UV-vis absorption measurements in dry chloroform.^58^ The radical anion exhibited a greenish-orange colour (Fig. S19a†) in solution and displayed the characteristic PDI radical anion peak at ∼684 nm in the UV-vis absorption spectra (Fig. S19b†). The ESA band at ∼575 nm obtained from the fsTA spectra of NO_2_-PDI in TOL (Fig. 5a-top) was compared to the radical anion UV-vis absorption peak of NO_2_-PDI, and it was confirmed that the long-lived excited-state observed in the fsTA as well as the nsTA spectra of NO_2_-PDI does not have any radical anion character.^59^

### Electron paramagnetic resonance (EPR) measurements

Solution state electron paramagnetic resonance (EPR) measurements of NO_2_-PDI were recorded in dry TOL and ACN at room temperature (RT, 298 K) under continuous light irradiation to probe the nature of the long-lived excited-state species. The EPR data of NO_2_-PDI in TOL (Fig. S20b†) and ACN (Fig. S20c†) do not exhibit the EPR peak characteristic of triplet or redox states of the PDI chromophore. The post-experiment colouration of the NO_2_-PDI sample (Fig. S21†) shows the photoconversion of NO_2_-PDI to ONO-PDI in ACN, while NO_2_-PDI was intact in TOL after the EPR experiment. EPR measurements of NO_2_-PDI were also recorded at liquid nitrogen temperatures (LNT, 77 K) in TOL and ACN. At LNT, the EPR spectrum of NO_2_-PDI did not show any characteristic peak in TOL nor in ACN (Fig. S22a and S22b†), which strongly substantiates the non-triplet and non-radical nature of the long-lived transient species involved in the photoisomerization of NO_2_-PDI to ONO-PDI in ACN.^48^

### Single wavelength nsTA decay kinetics studies

After establishing the non-triplet and non-radical nature of the long-lived species observed in the transient absorption spectra, we tried to explore the role of this long-lived species in the photorearrangement of NO_2_-PDI to ONO-PDI. To this purpose, a single-wavelength (420 nm) nsTA decay kinetics experiment was designed for NO_2_-PDI and executed in both ACN and TOL. The intensity of the ESA was monitored at 1 min intervals from 0 min to 40 min. The population–time profile of NO_2_-PDI in TOL (Fig. S23a†) showed negligible transformation of NO_2_-PDI throughout the experiment, as demonstrated by the almost equal intensity of the ESA at 0 min and 40 min (0 min, 20 min and 40 min nsTA traces are shown for better clarity and visibility). This observation was further strengthened by the typical UV-vis absorption spectra of NO_2_-PDI at 0 min and 40 min in TOL (Fig. S23b†). In contrast, NO_2_-PDI under the same experimental conditions in ACN evinced significant quenching of the ESA intensity when systematically probed from 0 min to 40 min (Fig. S24a†). The UV-vis absorption spectra of NO_2_-PDI in ACN at 0 min and 40 min revealed striking differences (Fig. S24b†). Firstly, at 0 min the UV-vis absorption spectrum was characterized by the typical absorption profile of NO_2_-PDI. At 40 min, considerable transformation of NO_2_-PDI to ONO-PDI was noted. Exponential fitting of the nsTA decay traces yielded congruent lifetimes for the 0 min (*τ* = 4.80 ± 0.04 ms), 20 min (*τ* = 4.90 ± 0.02 ms) and 40 min (*τ* = 4.90 ± 0.03 ms) traces in toluene (Fig. S25†). Moreover, the fitted lifetimes observed in ACN for the 0 min (*τ* = 6.90 ± 0.06 ms), 20 min (*τ* = 6.10 ± 0.02 ms) and 40 min (*τ* = 6.00 ± 0.02 ms) decay traces (Fig. S26†) present a small decrease in the lifetime of the long-lived species when compared to the lifetimes obtained in TOL. The above result serves as an additional proof, along with the quenching of the ESA intensity in ACN, for the involvement of the long-lived excited-state species in the photoisomerization of NO_2_-PDI.

### Computational investigations

**Fig. 6:**
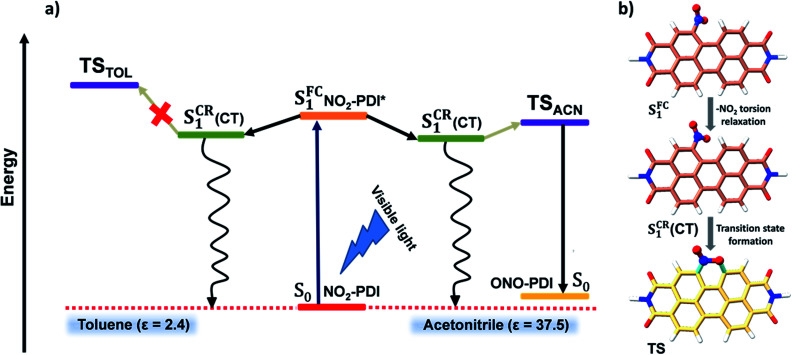
(a) A pictorial illustration presenting a plausible kinetic mechanism explaining the excited-state dynamics and associated photochemistry of NO_2_-PDI in the polar aprotic solvent acetonitrile and non-polar solvent toluene. Here, the S_0_ state represents the ground state of NO_2_-PDI and ONO-PDI, the S_1_^FC^ state represents the Franck–Condon state of NO_2_-PDI, and the S_1_^CR^(CT) state is a long-lived transient species observed in nsTA measurements, representing the conformationally relaxed singlet excited-state of NO_2_-PDI having charge-transfer character. (b) Optimized geometries showing the transition from the S_1_^FC^ state first to the S_1_^CR^(CT) state and finally to the six-membered TS through the nitro-aromatic torsion relaxation pathway computed at the CAM-B3LYP/6-311++G(d,p) level (the –R group was replaced by a –H atom to reduce the computational cost and increase clarity).

To understand the nature of the long-lived excited-state observed in the fsTA and nsTA measurements, we conducted theoretical and computational analyses for both NO_2_-PDI and ONO-PDI. Quantum chemical calculations using density functional theory (DFT) were carried out in the Gaussian 16 package to gain insights into the mechanistic details of the unprecedented photorearrangement of NO_2_-PDI to ONO-PDI. To account for the isomerization pathway, a six-membered transition state (TS) is proposed (Fig. 6 and S27†). The TS was located using quadratic synchronous transit-3 (QST3), optimized using transition state (TS-Berny) and verified using intrinsic reaction coordinate (IRC) methods. All the ground-state geometries were optimized at the CAM-B3LYP/6-311++G(d,p) level of theory (Fig. S28†). Single-point energy (SPE) calculations were performed on the ground-state geometries, and it was observed that the nitro (–NO_2_) isomer is more stable than the nitrito (–ONO) isomer by 0.12 kcal mol^−1^. The TS is located 77.60 kcal mol^−1^ higher in energy than the ground-state geometry of NO_2_-PDI. The relative ordering of the ground-state energies and optimized geometrical parameters of NO_2_-PDI, ONO-PDI and TS are illustrated in Fig. S29 and Table S7,† respectively. The relative stability of the linkage isomers was thoroughly investigated using Homolytic Bond Fragmentation (HBF) and Natural Bond Orbital (NBO) analyses. The C–O and C–N bond dissociation energies (BDE) were calculated using the HBF method in nitrito (–ONO) and nitro (–NO_2_) isomers, respectively. The C–O BDE was calculated to be 63.52 kcal mol^−1^, whereas the C–N BDE was found to be 56.98 kcal mol^−1^. The increase (Δ*E* = 6.54 kcal mol^−1^) in the BDE value from C–O to the C–N bond was supported by NBO analysis. The C–O bond (NBO energy ≅ −1.04 kcal mol^−1^) was estimated to be slightly more stable than the C–N bond (NBO energy ≅ −0.92 kcal mol^−1^). The stabilization energy *E*(2) correlated to the electron delocalization arising from the aromatic core to the π* acceptor orbital of NO_2_ (obtained from NBO calculations) was evaluated to be 3.91 kcal mol^−1^, while that arising from the aromatic core to the π* of ONO amounted to 1.07 kcal mol^−1^, computed at CAM-B3LYP/6-311++G(d,p). The above result affirmed that NO_2_ is a better π acceptor than ONO, and this further endorses the higher stability of the nitro isomer compared to the nitrito isomer, which was duly reflected in the single-point energy calculations (discussed earlier).^11,29^

The probable mechanism responsible for the photorearrangement of NO_2_-PDI to ONO-PDI could be an intramolecular rearrangement type mechanism,^60–62^ demonstrated in Fig. S30.† Deuterated ACN was used to confirm the intramolecular rearrangement mechanism of the photoisomerization reaction pathway. The deuterated product (Fig. S31a†) was not observed in the HRMS mass spectrum (Fig. S31b†), and therefore it was concluded that the photorearrangement of NO_2_-PDI to ONO-PDI most probably proceeds through an intramolecular rearrangement mechanism. The interactions (NBO) favouring the described mechanism are postulated in Table S8.† The lone pair, as well as the N–O bond pair in the nitro (–NO_2_) group, strongly interacts with the σ* orbital of the C8–H36 bond (reference structure given in Fig. S32†). The very high electron-withdrawing nature of the two imide groups in the PDI molecule renders the perylene core highly electron deficient, thereby subsequently increasing the electrophilicity of the core bay carbons, which can act as acceptors for nucleophilic substitution reactions. This donor–acceptor interaction is idealized as the first step towards the formation of a stable six-membered cyclic transition state, which is non-achievable in the case of other NA systems studied so far. The TS (i1549 cm^−1^ computed at CAM-B3LYP/6-311++G(d,p)) was located and verified using the IRC method in the Gaussian 16 package (Fig. S33†). Additionally, the IRC pathway predicted the dissociation of ONO-PDI into oxy-perylenediimide (PDI-O^˙^) and nitrosyl (NO^˙^) free radicals (Fig. S34†) below the ground-state minimum of ONO-PDI, accounting for the observed lower stability of the nitrito isomer.^11,63^

Electron localization function (ELF) isosurfaces were calculated to understand the electrostatic interactions present in the optimized geometry of the TS. The ELF plots and the respective cut planes are shown in Fig. S35.† The topologies of the molecular electron densities in Fig. S35a† delineate the polar covalent character of the partially formed O42–C8 bond in the TS. The electron density distribution around H46 in Fig. S35b† explains the partial bonding of the H atom with both the bay-position C atoms, C8 and C17, in the TS. Likewise, the 3D ELF isosurface (Fig. S35c†) reveals the partial bonding of O42–C8 and H46 to both the C8 and C17 perylene core carbon atoms, which further substantiates the bonding properties in the TS geometry. The bond order of the concerned six-membered TS (Table S9†) provided by the Wiberg bond indices is in good accordance with the calculated ELF isosurfaces. Molecular electrostatic surface potential (MESP) maps were computed for the optimized geometries of NO_2_-PDI, ONO-PDI and the TS in GaussView software. The NO_2_-PDI MESP map (Fig. S36†) displays an intermediate positive potential at the aromatic core compared to the lower positive potentials of TS and ONO-PDI. The electrostatic surfaces around the oxygen atoms in the nitro functionality (–NO_2_) in NO_2_-PDI exhibit large negative potential owing to the formation of a partial negative charge (*δ*^−^) on the oxygen atoms by virtue of resonance. The gradual decrease realized in the negative potential of the electrostatic surfaces around the oxygen atoms of the nitro group as we shift from NO_2_-PDI to ONO-PDI*via* the TS justifies the structural properties of the TS and product.

Intriguingly, we proceeded to inspect the involvement of a conformationally relaxed singlet excited-state (S^CR^_1_) in the photoisomerization of NO_2_-PDI. The importance of S^CR^_1_ states in the photochemistry of NAs has already been discussed in greater detail by Crespo-Hernández and co-workers.^33,64^ Motivated by their contributions, an *in silico* exploration of the excited-state analysis of NO_2_-PDI was pursued. The first singlet excited-state at the Franck–Condon geometry (S^FC^_1_) obtained through time-dependent density functional theory (TD-DFT) calculations was optimized, and SPE calculations were performed at the (CAM-B3LYP/6-311++G(d,p)) level of theory. The fully optimized S^CR^_1_ geometry was found to be stabilized by 6.22 kcal mol^−1^ with respect to the S^FC^_1_ geometry. The completely optimized geometrical parameters, along with the absolute energies of the S_0_, S^CR^_1_ and S^FC^_1_ states of NO_2_-PDI, are provided in Table S10.† The optimized S^CR^_1_ geometry was characterized by a nitro-aromatic torsion angle of 33.4°, which is significantly smaller than the nitro-aromatic torsion angle of 59.9° observed in the ground-state optimized geometry of NO_2_-PDI (Fig. 6b and S37†). It is well known in the literature that the conformational relaxation of NO_2_-PDI*via* the nitro-aromatic torsion angle serves as a reaction coordinate for the deactivation of the molecule from the initially populated excited states.^65,66^ The extent of conformational relaxation in various dielectric environments was further probed in the solvents ACN (*ε* = 37.5) and TOL (*ε* = 2.4). The S^FC^_1_ geometry was optimized in the presence of ACN and TOL, respectively, using the polarizable continuum (PCM) model at the CAM-B3LYP/6-311++G(d,p)) level of theory. The S^CR^_1_ optimized geometry in the ACN solvent model showed higher conformational relaxation with a nitro-aromatic torsion angle of 31.3°, whereas in the TOL solvent model the nitro-aromatic torsion angle was observed to be 32.7°. This result confirms the tendency of the nitro group to attain a more parallel-like orientation with respect to the aromatic core in the excited-state, which was enhanced in polar solvents like ACN.

The underlying charge-transfer (CT) character of the S^CR^_1_ state was scrutinized using TD-DFT and frontier molecular orbital (FMO) isosurface analyses. Vertical excitation energy calculations were carried out with the S^CR^_1_ optimized geometry of NO_2_-PDI, and it was noted that the first singlet excited (S_1_) state is composed of a 100% HOMO → LUMO transition with an oscillator strength (*f*) of 0.6270. With this observation, we moved forward to visualize the electron density distribution in the HOMO and LUMO level (the molecular orbitals were drawn using an isovalue of 0.02 au, Fig. S38†). The electron density in the nitro group is noticeably larger in the LUMO than in the HOMO, suggesting a partial CT character in the S^CR^_1_ state of NO_2_-PDI.^53,67–69^ Another interesting observation from the FMO isosurface diagrams is that the electron density of the nitro group, specifically the lone pair (LP) on the oxygen atom in the LUMO (Fig. S39†) interacts with the C–H bond present in the adjacent bay position of the perylene core. This interaction is probably a LP(O) → σ*(C–H) type of interaction, and serves as strong evidence for the earlier proposed six-membered TS.^70^ Together with the aid of spectroscopic signatures and computational evidence, we anticipate that the long-lived species observed in the fsTA and nsTA spectra of NO_2_-PDI is the conformationally relaxed singlet excited-state (S^CR^_1_), featuring an unusually long lifetime (∼4.80–6.90 ms), which is reasonably rationalized by the partial CT character of the S^CR^_1_ state. Previously, it has been shown that photochemical reactions on millisecond timescales could be important for developing novel negative fast photochromic materials as well as for the development of advanced photofunctional materials in the field of nonlinear optics.^71,72^

Fig. 6a displays a consolidated representation of the proposed energy level diagram that effectively incorporates the entire scheme of this work. Initially, the S^FC^_1_ state is populated upon photoexcitation of NO_2_-PDI with visible light. The S^FC^_1_ state decays *via* conformational relaxation of the NO_2_-PDI S^FC^_1_ geometry to give rise to the new state S^CR^_1_, which is long-lived and has moderate CT character. The newly formed S^CR^_1_ state bifurcates into two non-radiative relaxation channels. The primary relaxation channel is the non-radiative deactivation to the ground state (S_0_). The second minor channel connects the S^CR^_1_ state to the six-membered TS, through which product formation finally takes place. The S^CR^_1_ state is populated in both the solvents TOL and ACN, and the population of the TS is the most crucial step responsible for the observed photochemistry in NO_2_-PDI. The TS was optimized in the presence of ACN (TS_ACN_) and TOL (TS_TOL_), respectively, using the polarizable continuum (PCM) model available under the (CAM-B3LYP/6-311++G(d,p)) level of theory. The optimized TS_ACN_ geometry was computed to be substantially stabilized compared to the TS_TOL_ geometry, with an energy difference of Δ*E* = 5.77 kcal mol^−1^, supported by the moderate increase in the calculated dipole moment values of the optimized transition state in the presence of ACN relative to TOL (Table S11†). The computational analysis of the TS energy stabilization in ACN and TOL is in good agreement with our experimental results, in which we observed that the rate of photoisomerization of NO_2_-PDI in the presence of ACN is exceptionally higher than that in TOL, as validated from the integration of TD laser irradiation, single wavelength nsTA decay kinetics and TD-DFT computational studies.

Certainly, a more comprehensive analysis of the nature of the long-lived species observed in the ultrafast spectroscopic experiments awaits the results of molecular dynamics (MD) simulations and multiconfigurational self-consistent-field (MCSCF) calculations to understand the specificity of a particular perylenediimide (nitro-perylenediimide) in favouring facile photoisomerization in polar aprotic solvents.

## Conclusions

In conclusion, we report the first experimental evidence for the existence of linkage isomerism in a perylenediimide chromophore. Nitrito-perylenediimide has been isolated from the visible-light excitation of NO_2_-PDI in ACN and unequivocally characterized using state-of-the-art techniques, including XPS, FTIR, Raman, ^1^H-NMR, CHN, and HRMS analyses. The experimentally observed lower stability of ONO-PDI compared to NO_2_-PDI was well supported by SPE, NBO, and IRC quantum chemical calculations. The photoisomerization process was ascertained to be highly sensitive to the dielectric properties of the solvent. Time-dependent laser irradiation experiments, in addition to single-wavelength nsTA decay kinetics studies, confirmed the favourability of photoisomerization in a highly polar aprotic solvent like acetonitrile compared to that in non-polar toluene. fsTA and nsTA experiments provided crucial information about the excited-state dynamics of NO_2_-PDI in solution, in which the formation of a long-lived species was evinced after the decay of the initially populated singlet excited-state. The non-triplet and non-radical character of the long-lived excited-state were meticulously probed using standard triplet–triplet energy transfer and chemical reduction, followed by UV-vis absorption and temperature-dependent EPR studies. The nitro-aromatic torsion-angle-based conformationally relaxed singlet excited-state manifesting modest CT character is rationalized as the long-lived excited-state observed in ultrafast TA experiments. Through extensive computational investigations, a six-membered cyclic transition state has been proposed, explaining the plausible pathway of the observed photoisomerization. The TS was calculated to be appreciably stabilized in ACN in comparison to TOL, which is most evidently responsible for the atypical photoisomerization of NO_2_-PDI in a polar aprotic solvent. Finally, through the combination of spectroscopic and computational evidence, a unified kinetic mechanism was proposed to elucidate the excited-state dynamics and correlated photochemistry of NO_2_-PDI.

Further advanced investigations based on two-dimensional spectroscopy and multiconfigurational high-level *ab initio* calculations should be able to provide a more detailed picture of the photoisomerization process and the nature of the excited states involved therein. Additionally, time-resolved infrared and Raman spectroscopy could prove to be of immense importance in obtaining a real-time understanding of the conformational relaxations observed in nitroaromatics.

## Data availability

All experimental/computational data and procedures are available in the ESI.†

## Author contributions

A. M., E. S., and M. H. conceived the project; A. M. and E. S. carried out the measurements; A. M. carried out the calculations. A. M. and E. S. analysed the results; A. M., E. S., and M. H. wrote the manuscript; M. H. supervised the research.

## Conflicts of interest

There are no conflicts to declare.

## Supplementary Material

SC-013-D2SC02979K-s001

## References

[cit1] Burmeister J. L. (1968). Coord. Chem. Rev..

[cit2] Naumov P., Sahoo S. C., Zakharov B. A., Boldyreva E. V. (2013). Angew. Chem., Int. Ed..

[cit3] Grenthe I., Nordin E. (1979). Inorg. Chem..

[cit4] Tishkov A. A., Schmidhammer U., Roth S., Riedle E., Mayr H. (2005). Angew. Chem., Int. Ed..

[cit5] Ciofini I., Adamo C. (2001). J. Phys. Chem. A.

[cit6] Balzani V., Ballardini R., Sabbatini N., Moggi L. (2002). Inorg. Chem..

[cit7] Benmansour S., Setifi F., Triki S., Gómez-García C. J. (2012). Inorg. Chem..

[cit8] Nilsson Z. N., Mandella B. L., Sen K., Kekilli D., Hough M. A., Moënne-Loccoz P., Strange R. W., Andrew C. R. (2017). Inorg. Chem..

[cit9] Coppens P., Novozhilova I., Kovalevsky A. (2002). Chem. Rev..

[cit10] Ju K.-S., Parales R. E. (2010). Microbiol. Mol. Biol. Rev..

[cit11] Rodríguez-Córdoba W., Gutiérrez-Arzaluz L., Cortés-Guzmán F., Peon J. (2021). Chem. Commun..

[cit12] Mohammed O. F., Vauthey E. (2008). J. Phys. Chem. A.

[cit13] Kovalenko S. A., Schanz R., Farztdinov V. M., Hennig H., Ernsting N. P. (2000). Chem. Phys. Lett..

[cit14] Thomsen C. L., Thøgersen J., Keiding S. R. (1998). J. Phys. Chem. A.

[cit15] Ieda N., Nakagawa H., Horinouchi T., Peng T., Yang D., Tsumoto H., Suzuki T., Fukuhara K., Miyata N. (2011). Chem. Commun..

[cit16] Salgo M. G., Stone K., Squadrito G. L., Battista J. R., Pryor W. A. (1995). Biochem. Biophys. Res. Commun..

[cit17] Wink D. A., Kasprzak K. S., Maragos C. M., Elespuru R. K., Misra M., Dunams T. M., Cebula T. A., Koch W. H., Andrews A. W., Allen J. S., Keefer L. K. (1991). Science.

[cit18] Nguyen T., Brunson D., Crespi C. L., Penman B. W., Wishnok J. S., Tannenbaum S. R. (1992). Proc. Natl. Acad. Sci. U. S. A..

[cit19] Marvin-Sikkema F. D., de Bont J. A. M. (1994). Appl. Microbiol. Biotechnol..

[cit20] Wilson J., Octaviani M., Bandowe B. A. M., Wietzoreck M., Zetzsch C., Pöschl U., Berkemeier T., Lammel G. (2020). Environ. Sci. Technol..

[cit21] García-Berríos Z. I., Arce R. (2012). J. Phys. Chem. A.

[cit22] Hamanoue K., Amano M., Kimoto M., Kajiwara Y., Nakayama T., Teranishi H. (2002). J. Am. Chem. Soc..

[cit23] Sebastian E., Philip A. M., Benny A., Hariharan M. (2018). Angew. Chem., Int. Ed..

[cit24] Lijina M. P., Benny A., Ramakrishnan R., Nair N. G., Hariharan M. (2020). J. Am. Chem. Soc..

[cit25] Nagarajan K., Mallia A. R., Muraleedharan K., Hariharan M. (2017). Chem. Sci..

[cit26] Sasikumar D., John A. T., Sunny J., Hariharan M. (2020). Chem. Soc. Rev..

[cit27] Madhu M., Ramakrishnan R., Vijay V., Hariharan M. (2021). Chem. Rev..

[cit28] Sebastian E., Hariharan M. (2022). ACS Energy Lett..

[cit29] Muya J. T., Chung H., Lee S. U. (2018). RSC Adv..

[cit30] Belligund K., Mathew T., Hunt J. R., Nirmalchandar A., Haiges R., Dawlaty J., Prakash G. K. S. (2019). J. Am. Chem. Soc..

[cit31] Giussani A., Worth G. A. (2017). J. Chem. Theory Comput..

[cit32] Hause M. L., Herath N., Zhu R., Lin M. C., Suits A. G. (2011). Nat. Chem..

[cit33] Vogt R. A., Reichardt C., Crespo-Hernández C. E. (2013). J. Phys. Chem. A.

[cit34] Zhang W., Song H., Kong J., Kuang Z., Li M., Guo Q., Chen C. F., Xia A. (2019). J. Phys. Chem. C.

[cit35] Kuang Z., He G., Song H., Wang X., Hu Z., Sun H., Wan Y., Guo Q., Xia A. (2018). J. Phys. Chem. C.

[cit36] Chapman O. L., Heckert D. C., Reasoner J. W., Thackaberry S. P., Battegay M., Brandt P., Moritz J., Griswold A. A., Hoganson E., Lenz G. (2002). J. Am. Chem. Soc..

[cit37] Chen K.-Y., Fang T.-C., Chang M.-J. (2012). Dyes Pigm..

[cit38] Bartle K. D., Perry D. L., Wallace S. (1987). Fuel Process. Technol..

[cit39] Zakharova I. A., Salyn J. V., Tatjanenko L. V., Mashkovsky Y. S., Ponticelli G. (1981). J. Inorg. Biochem..

[cit40] Bandara H. M. D., Burdette S. C. (2012). Chem. Soc. Rev..

[cit41] Penland R. B., Lane T. J., Quagliano J. V. (2002). J. Am. Chem. Soc..

[cit42] Hatcher L. E., Skelton J. M., Warren M. R., Raithby P. R. (2019). Acc. Chem. Res..

[cit43] Passingham C., Hendra P. J., Hodges C., Willis H. A. (1991). Spectrochim. Acta Part A Mol. Spectrosc..

[cit44] Zobel J. P., Heindl M., Nogueira J. J., González L. (2018). J. Chem. Theory Comput..

[cit45] Buckup T., Kraack J. P., Marek M. S., Motzkus M. (2013). EPJ Web Conf..

[cit46] Gutiérrez-Moreno D., Sastre-Santos A., Fernández-Lázaro F. (2018). Org. Chem. Front..

[cit47] Due to the low solubility of **ONO-PDI** in low and high polar solvents, we have chosen moderately polar chloroform solvent for the steady-state optical measurements of **NO**_**2**_**-PDI** and **ONO-PDI**. To unravel the dielectric constant dependent photoisomerization mechanism of **NO**_**2**_**-PDI**, ultrafast transient absorption measurements, laser photoirradiation studies and electron paramagnetic resonance measurements have been performed in low polar toluene (*ε* = 2.4) and highly polar acetonitrile (*ε* = 37.5) solvent

[cit48] Sharma V., Puthumana U., Karak P., Koner A. L. (2018). J. Org. Chem..

[cit49] Laskar I. R., Das D., Mostafa G., Lu T. H., Keng T. C., Wang J. C., Ghosh A., Chaudhuri N. R. (2001). New J. Chem..

[cit50] Hatcher L. E., Bigos E. J., Bryant M. J., MacCready E. M., Robinson T. P., Saunders L. K., Thomas L. H., Beavers C. M., Teat S. J., Christensen J., Raithby P. R. (2014). CrystEngComm.

[cit51] Gegiou D., Muszkat K. A., Fischer E. (2002). J. Am. Chem. Soc..

[cit52] Snellenburg J. J., Laptenok S., Seger R., Mullen K. M., van Stokkum I. H. M. (2012). J. Stat. Software.

[cit53] Brister M. M., Piñero-Santiago L. E., Morel M., Arce R., Crespo-Hernández C. E. (2017). J. Phys. Chem. A.

[cit54] Sebastian E., Hariharan M. (2021). J. Am. Chem. Soc..

[cit55] Mohan A., Sebastian E., Gudem M., Hariharan M. (2020). J. Phys. Chem. B.

[cit56] Schierl C., Niazov-Elkan A., Shimon L. J. W., Feldman Y., Rybtchinski B., Guldi D. M. (2018). Nanoscale.

[cit57] Lower S. K., El-Sayed M. A. (2002). Chem. Rev..

[cit58] Banthorpe D. V., Charlwood B. V., Wassermann A., Horsfield A. (1972). J. Chem. Soc., Chem. Commun..

[cit59] Spenst P., Young R. M., Wasielewski M. R., Würthner F. (2016). Chem. Sci..

[cit60] Kovalevsky A. Y., King G., Bagley K. A., Coppens P. (2005). Chem. - Eur. J..

[cit61] Giussani A. (2014). J. Chem. Theory Comput..

[cit62] Watanabe Y., Tanaka T., Fukuyoshi S., Oda A. (2015). J. Photochem. Photobiol., A.

[cit63] Hamanoue K., Nakayama T., Amijima Y., Ibuki K. (1997). Chem. Phys. Lett..

[cit64] Vogt R. A., Crespo-Hernández C. E. (2013). J. Phys. Chem. A.

[cit65] Rafiq S., Yadav R., Sen P. (2011). J. Phys. Chem. A.

[cit66] Ghosh R., Palit D. K. (2012). J. Phys. Chem. A.

[cit67] Collado-Fregoso E., Zugazagoitia J. S., Plaza-Medina E. F., Peon J. (2009). J. Phys. Chem. A.

[cit68] Brister M. M., Piñero-Santiago L. E., Morel M., Arce R., Crespo-Hernández C. E. (2016). J. Phys. Chem. Lett..

[cit69] Imahori H., Kobori Y., Kaji H. (2021). Acc. Chem. Res..

[cit70] Slavov C., Yang C., Heindl A. H., Wegner H. A., Dreuw A., Wachtveitl J. (2020). Angew. Chem., Int. Ed..

[cit71] Yamaguchi T., Kobayashi Y., Abe J. (2016). J. Am. Chem. Soc..

[cit72] Kobayashi Y., Abe J. (2022). Chem. Soc. Rev..

