# Supplementary material for: Solvent dielectric delimited nitro–nitrito photorearrangement in a perylenediimide derivative
Source: Chem Sci. 2022 Jul 4;13(30):8860–70. doi: 10.1039/d2sc02979k (PMC9350666; doi:10.1039/d2sc02979k)
Supplement: SC-013-D2SC02979K-s001 [file SC-013-D2SC02979K-s001.pdf]

Electronic Supplementary Material (ESI) for Chemical Science.
This journal is © The Royal Society of Chemistry 2022

Solvent Dielectric Delimited Nitro-Nitrito Photorearrangement in a Perylenediimide Derivative

Aniruddha Mazumder, Ebin Sebastian and Mahesh Hariharan*[a]

[a]School of Chemistry, Indian Institute of Science Education and Research Thiruvananthapuram (IISER TVM), Maruthamala P. O., Vithura, Thiruvananthapuram695551, Kerala, India.

*E-mail:mahesh@iisertvm.ac.in

Supporting Information (SI)

Table of Contents

Section 1: Materials and Methods .... SI 5

1.1 Computational Analysis........ SI 5

1.2 Femtosecond Transient Absorption (fsTA) Measurement........ SI 5

1.3 Nanosecond Transient Absorption (nsTA) Measurement........ SI 6

1.4 Global Analysis .... SI 6

1.5 Fourier-Transform Infrared Spectroscopic (FTIR) Analysis........ SI 6

1.6 Raman Spectroscopic Analysis........ SI 6

1.7 X-ray Photoelectron Spectroscopic (XPS) Analysis........ SI 6

1.8 Photoirradiation Experiments........ SI 6

1.9 Electron Paramagnetic Resonance (EPR) Experiments........ SI 6

Section 2: Syntheses and Characterization........ SI 7

Scheme S1: Showing the synthesis of PDI, NO_2-PDI, and ONO-PDI .... SI 7

2.1 Synthesis of PDI........ SI 7

2.2 Synthesis of NO_2-PDI........ SI 7

2.3 Synthesis of ONO-PDI .... SI 8

Section 3: Tables........ SI 9

Table S1: Selected publications showing the evolution of nitrite linkage isomers reported over the years........ SI 9

Table S2: NO_2-PDI and ONO-PDI CHN elemental analysis (corrected with hexane as solvent)........ SI 9

Table S3: Oscillator strength for different transitions of NO_2-PDI and ONO-PDI calculated at TD-CAM-B3LYP/ 6-311++G(d,p) level of theory........ SI 10

Table S4: UV-vis and fluorescence spectroscopic data of NO_2-PDI and ONO-PDI measured in toluene at room temperature........ SI 10

Table S5: Lowest-energy triplet states of nitro-perylenediimide (NO_2-PDI) and the respective triplet quenchers used. ……SI 11

Table S6: Spin-orbit coupling values between four bright states (S_1, S_2, S_3, S_4) and low lying triplet states at NO_2-PDI minimum evaluated at TD-CAM-B3LYP/6-311++G(d,p) level of theory using PySOC package implemented in Gaussian 16……SI 11

Table S7: Ground state (S_0) optimized geometrical parameters of NO_2-PDI, ONO-PDI and TS at CAM-B3LYP/6-311++G(d,p) level of theory……SI 12

Table S8: Natural Bond Orbital (NBO) interactions supporting the proposed intramolecular mechanism of photoisomerization of NO_2-PDI to ONO-PDI evaluated at CAM-B3LYP/6-311 ++G(d,p) level of theory……SI 12

Table S9: Theoretical Bond Order of selected bonds in the proposed 6-membered TS calculated from the Wiberg Bond Indices evaluated in Multiwfn 3.8……SI 13

Table S10: Completely optimized geometrical parameters along with the absolute energies of ground-state (S_0), relaxed singlet excited-state (S_1^{CR}) and singlet Franck-Condon state (S_1^{FC}) of NO_2-PDI computed at CAM-B3LYP/6-311++G(d,p) level of theory……SI 13

Table S11: Calculated dipole moment values of the transition state in vacuum, polar acetonitrile and non-polar toluene solvent models at CAM-B3LYP/6-311++G(d,p)) level of theory……SI 13

Section D: Figures……SI 14

Figure S1: a) Transformation of NO_2-PDI to yellow coloured ONO-PDI under visible light irradiation for 2 hrs in acetonitrile. b) TLC showing red coloured NO_2-PDI and yellow coloured ONO-PDI (DCM:EtOAc=2:1)……SI 14

Figure S2: (Top) Core level C (1S) XPS spectrum of a) NO_2-PDI and b) ONO-PDI. (Bottom) Core level O (1S) spectrum of c) NO_2-PDI and d) ONO-PDI……SI 14

Figure S3: Raman spectra of a) NO_2-PDI and b) ONO-PDI measured in the powder state after photoexcitation at 632.8 nm……SI 15

Figure S4: ^{1}H-NMR spectrum of NO_2-PDI in $CDCl_3$. Inset a) shows the splitting of the core aromatic protons……SI 15

Figure S5: ^{1}H-NMR spectrum of ONO-PDI in $CDCl_3$. Inset a) shows the splitting of the core aromatic protons……SI 16

Figure S6: Superimposed ^{1}H-NMR spectra of NO_2-PDI and ONO-PDI in $CDCl_3$. Inset a) shows the splitting of the core aromatic protons……SI 16

Figure S7: HRMS (APCI) spectra of a) NO_2-PDI and b) ONO-PDI in $CHCl_3$……SI 17

Figure S8: a) Normalized absorption spectra of PDI (black line) and NO_2-PDI (red line), b) Emission spectra of PDI (black line) and NO_2-PDI (red line) in $CHCl_3$ at room temperature. Zoomed inset c) Showing the normalized emission of PDI (black line) and NO_2-PDI x 345 units (red line)……SI 17

Figure S9: Normalized absorption (solid black line) and emission spectra (dotted red line) of a) NO_2-PDI and b) ONO-PDI in $CHCl_3$ at room temperature……SI 18

Figure S10: Resonant Raman spectrum of NO_2-PDI measured in the powder state upon excitation at 488 nm with an acquisition time of 5 s using a 50x objective……SI 18

Figure S11: NO_2-PDI solutions before and after heating along with the UV-vis absorption spectrum in a) acetonitrile and b) toluene...SI 19

Figure S12: a) nsTA (λ_{ex} = 532 nm) spectra of NO_2-PDI in acetonitrile. b) Decay trace showing excited-state absorption at 420 nm obtained from nsTA measurements of NO_2-PDI in acetonitrile.SI 19

Figure S13: (Top) nsTA features of NO_2-PDI in nitrogen-purged toluene solution at a) 400 nm, b) 420 nm and c) 450 nm. (Bottom) Triplet quenching studies of NO_2-PDI in oxygen-purged toluene solution at a) 400 nm, b) 420 nm and c) 450 nm……………………………………………………………….SI 20

Figure S14: a) nsTA features of NO_2-PDI in nitrogen-purged toluene solution at 400 nm. b) Triplet-triplet energy transfer studies of NO_2-PDI using 250 μL 2M β-carotene at 420 nm………………....SI 20

Figure S15: (Left) nsTA features of NO_2-PDI in nitrogen-purged toluene solution at 420 nm. (Right) Triplet-triplet energy transfer studies of NO_2-PDI probed at 420 nm using 250 μL 2M a) Anthracene, b) Naphthalene and c) Perylene………………...SI 21

Figure S16: (Left) nsTA features of NO_2-PDI in nitrogen-purged toluene solution at 420 nm. (Right) Triplet-triplet energy transfer studies of NO_2-PDI probed at 420 nm using 250 μL 2M d) Ferrocene, e) Fullerene-C_{60} and f) 1-Bromopyrene…………………………………………………………………...SI 22

Figure S17: Temperature-dependent nsTA spectra of NO_2-PDI in a) toluene and b) acetonitrile. N. B.- At lower temperatures the lifetime of the NO_2-PDI transient feature increases and the decay of this transient species is not complete. The most probable reason for the increase in the lifetime of the transient species is the rigidification of the molecule at lower temperatures, thereby reducing the total degrees of freedom and suppression of the deactivation channels of NO_2-PDI……………………..SI 23

Figure S18: Temperature-dependent nsTA spectra of NO_2-PDI in toluene, showing the decay traces at room temperature, -10° C and slow warming to room temperature conditions. N. B.- The persistence of the long-lived transient feature in toluene at room temperature, -10° C and slow warming to room temperature conditions clearly demonstrates the excited-state photophysical transformation of the NO_2-PDI molecule and rules out the possibility of the observed long-lived excited-state to be a photochemical species…………………………………………………………….SI 24

Figure S19: a) NO_2-PDI radical anion solution after cobaltocene addition. b) UV-vis absorption spectra of NO_2-PDI before and after addition of cobaltocene in $CHCl_3$……………………………....SI 24

Figure S20: Electron paramagnetic resonance (EPR) spectrum of a) Empty sample capillary, b) NO_2-PDI in toluene solvent and c) NO_2-PDI in acetonitrile solvent recorded at room temperature under continuous light irradiation. N. B.- At room temperature no prominent peak was observed in the NO_2-PDI EPR spectrum in toluene and acetonitrile, and only background noise from the sample capillary was observed……………………………………………………………………………………………..SI 25

Figure S21: NO_2-PDI sample after EPR measurements under continuous light irradiation conditions at room temperature in a) toluene and b) acetonitrile solvents. N. B.- Red colour of NO_2-PDI sample was intact in toluene after EPR experiment, whereas red coloured NO_2-PDI transformed to yellow coloured ONO-PDI after EPR experiment in acetonitrile……………………………………………….SI 25

Figure S22: Electron paramagnetic resonance (EPR) spectrum of a) NO_2-PDI in toluene and b) NO_2-PDI in acetonitrile solvents recorded at liquid nitrogen temperature…………………………………SI 25

Figure S23: a) Single wavelength (420 nm) nsTA decay kinetics of NO_2-PDI in toluene at 0 min, 20 min and 40 min. b) UV-vis absorption spectra of NO_2-PDI at 0min and 40 min in toluene………..SI 26

Figure S24: a) Single wavelength (420 nm) nsTA decay kinetics of NO_2-PDI in acetonitrile at 0 min, 20 min and 40 min. b) UV-vis absorption spectra of NO_2-PDI at 0min and 40 min in acetonitrile....SI 26

Figure S25: Exponentially fitted decay lifetime of the excited-state absorption of NO_2-PDI in toluene at a) 0 min, b) 20 min and c) 40 min obtained through the single wavelength (nsTA) decay kinetics experiment……………………………………………………………………………………………..SI 27

Figure S26: Exponentially fitted decay lifetime of the excited-state absorption of NO_2-PDI in acetonitrile at a) 0 min, b) 20 min and c) 40 min obtained through the single wavelength (nsTA) decay kinetics experiment…………………………………………………………………………………....SI 27

Figure S27: Optimized 6-membered transition state geometry at CAM-B3LYP/6-311++G(d,p) level of theory……………………………………………………………………………………………………...SI 27

Figure S28: a) NO_2-PDI and b) ONO-PDI optimized geometries at CAM-B3LYP/6-311++G(d,p) level of theory……………………………………………………………………………………………..SI 28

Figure S29: Relative energy ordering of NO_2-PDI, Transition state and ONO-PDI. Energies calculated with the respective optimized geometries at CAM-B3LYP/6-311++G(d,p) level of theory……….....SI 28

Figure S30: Plausible intramolecular rearrangement mechanism of NO_2-PDI photoisomerization to ONO-PDI…………………………………………………………………………………………......SI 29

Figure S31: a) Transformation of NO_2-PDI to yellow coloured ONO-PDI under visible light irradiation for 2 hrs in deuterated acetonitrile (CD_3CN). b) HRMS spectrum showing the presence of non-incorporated deuterium product…………………………………………………………………...SI 29

Figure S32: NO_2-PDI structure considered for Natural Bonding Orbital (NBO) calculations (3-aminopentane group is replaced by H atom to reduce computational cost)…………………………SI 29

Figure S33: Intrinsic reaction coordinate (IRC) pathway of photoisomerization of NO_2-PDI to ONO-PDI calculated at CAM-B3LYP/6-311++G(d,p) level of theory……………………………………..SI 30

Figure S34: Intrinsic reaction coordinate (IRC) pathway showing the dissociation of ONO-PDI into nitrosyl and aryloxy free radicals below the ONO-PDI minimum computed at CAM-B3LYP/6-311++G(d,p) level of theory………………………………………………………………………………SI 30

Figure S35: 2D-Electron localization function (ELF) plots of a) TS showing bond formation betweeen O42-C8 atoms b) TS showing the bond formation between C17-H46 and bond breaking between C8-H46 c) 3D-ELF plot of the TS..SI 31

Figure S36: Molecular electrostatic potential (MESP) maps computed at the optimized geometries of NO_2-PDI, Transition state and ONO-PDI with CAM-B3LYP/6-311++G(d,p) level of theory…..........SI 31

Figure S37: Conformationally relaxed singlet excited-state optimized geometry of NO_2-PDI showing a) Top view, b) Side view and c) Ground-state optimized geometry of NO_2-PDI at CAM-B3LYP/6-311++G(d,p) level of theory………………………………………………………………………………SI 32

Figure S38: a) HOMO-LUMO isosurfaces (isovalue 0.02 a.u.) computed from the TD-DFT calculation with S_0 optimized geometry of NO_2-PDI and b) HOMO-LUMO isosurfaces (isovalue 0.02 a.u.) computed from the TD-DFT calculation with S_1 optimized geometry of NO_2-PDI at CAM-B3LYP/6-311++G(d,p) level of theory……………………………………………………………….....................SI 32

Figure S39: LUMO isosurface (isovalue 0.02 a.u.) visualized from the TD-DFT calculation with S_1 optimized geometry of NO_2-PDI. Zoomed inset a) showing the interactions between O (lone pair) and C-H orbitals in conformationally relaxed singlet excited-state of NO_2-PDI…………………………...SI 33

Section 1: Materials and Methods

All chemicals were obtained from commercial suppliers and used as received without further purification. All reactions were carried out in oven-dried glassware prior to use. Solvents were dried and distilled by standard laboratory purification techniques. TLC analyses were performed on recoated aluminum plates of silica gel 60 F254 plates (0.25 mm, Merck), and developed TLC plates were visualized under short and long-wavelength UV lamps. Flash column chromatography was performed using silica gel of 200-400 mesh employing a solvent polarity correlated with the TLC mobility observed for the substance of interest. Yields refer to chromatographically and spectroscopically homogenous substances. Melting points were obtained using a capillary melting point apparatus. ^{1}H and ^{13}C NMR spectra were measured on a 500 MHz Bruker advance DPX spectrometer. The internal standard used for ^{1}H and ^{13}C NMR is tetramethylsilane (TMS). High-resolution mass spectra (HRMS) were recorded on Thermo scientific Q exactive mass spectrometer using the Atmospheric pressure chemical ionization (APCI, positive mode) technique. Photophysical measurements of the derivatives were carried out in a cuvette of 3 mm path length. Absorption and emission spectra were recorded on Shimadzu UV-3600 UV-VIS-NIR and Horiba Jobin Yvon Fluorolog spectrometers, respectively.

1.1 Computational Analysis

All the calculations are carried out in Gaussian 16 employing the CAM-B3LYP functional and 6-311++G(d,p) basis set at the DFT level of theory in vacuum unless stated otherwise. Vertical excitation energies and oscillator strengths were calculated employing time-dependent DFT (TD-DFT) at the CAM-B3LYP functional and 6-311++G(d,p) level of theory. The frontier molecular orbitals (FMO) of NO_2-PDI was obtained from the generated cube files of energy calculations, high quality FMO isosurfaces were created using GaussView 5.0.8 software. Geometry optimizations and single point energy (SPE) calculations were carried out using CAM-B3LYP functional and 6-311++G(d,p) basis set at the DFT level of theory in Gaussian 16[1]. Transition state (TS) analysis was done using quadratic synchronous transit-3 (QST3), optimized using transition state (TS-Berny) and verified using intrinsic reaction coordinate (IRC) methods. Homolytic Bond Fragmentation (HBF) and Natural Bond Orbital (NBO) analyses were performed on the optimized geometry of NO_2-PDI. Electron localization function (ELF) isosurfaces along with the molecular electrostatic surface potential (MESP) maps were calculated for the optimized TS geometry in Multiwfn 3.8[2] and GaussView 5.0.8[3] softwares respectively. SOC values were calculated using PySOC[4] package implemented in Gaussian.

1.2 Femtosecond Transient Absorption (fsTA) Measurement

A Spectra-Physics Mai Tai SP mode-locked laser (86 MHz, 800 nm) was used as a seed for a Spectra-Physics Spitfire ace regenerative amplifier (1 kHz, 5.5 mJ). A fraction of the amplified output was used to produce a 440 nm pump pulse by TOPAS. A residual pulse of 800 nm was sent through an optical delay line inside an ExciPro pump-probe spectrometer to produce a white light continuum by employing a sapphire crystal. The white light continuum was split into two, and the streams were used as a probe and reference pulses. The femtosecond transient absorption spectra of the sample were recorded using a dual diode array detector, having a 200 nm detection window and 3.5 ns optical delay. Sample solutions were prepared in a rotating cuvette with a 1.2 mm path length. Determination of an appropriate instrument response function (IRF) is needed for accurate deconvolution of recorded transient absorption data. The IRF was determined by a solvent (10% benzene in methanol) two-photon absorption and was found to be ~110 fs at about 530 nm. A neutral density filter (80%) was used for controlling the incident flux on the sample. fsTA measurement of NO_2-PDI in toluene solvent was recorded by photoexciting the sample with 470 nm, 200 nJ, and 100 fs pulses to moderate singlet–singlet annihilation that often arises in multi-chromophoric assemblies[5]. The observed kinetic components were laser intensity-independent, ruling out the chance of singlet–singlet annihilation. fsTA measurements of NO_2-PDI in acetonitrile were recorded in low laser fluence and shorter acquisition time to avoid any undesired photoisomerization/degradation of the NO_2-PDI sample during measurement.

1.3 Nanosecond Transient Absorption (nsTA) Measurement

Nanosecond laser flash photolysis experiments of the nitrogen-purged solution of NO_2-PDI in toluene and acetonitrile was done in an Applied Photophysics Model LKS-60 laser kinetic spectrometer using the third harmonic (532 nm, pulse duration ≈10 ns) of a Quanta Ray INDI-40-10 series pulsed Nd:YAG laser as the excitation source. In acetonitrile, the measurement was completed under low fluence (laser power 5) to avoid unwanted rapid photodegradation/photodissociation. Exponential fitting of the decay traces obtained from the single wavelength (420 nm) nsTA decay kinetics experiment was performed with the help of OriginPro software[6].

1.4 Global Analysis

Global analyses of the fsTA and nsTA spectra were performed using the Glotaran software[7]. The procedure evaluates the instrument time response function and group velocity dispersion of the white continuum and allows one to compute decay time constants and dispersion-compensated spectra. In global analysis, all the wavelengths were analyzed concurrently, employing a sequential model to give species associated difference spectra (SAS). The SAS indicate that the evolution of the spectra in time and do not necessarily denote a real physical/chemical species. SAS designate the spectral changes that occur with their associated time constants.

1.5 Fourier-Transform Infrared Spectroscopic (FTIR) Analysis

Fourier transform infrared spectroscopy (FTIR) measurements of NO_2-PDI and ONO-PDI were recorded on a Shimadzu IR Prestige-21 FTIR spectrometer as KBr pellets.

1.6 Raman Spectroscopic Analysis

Raman spectroscopic analysis of NO_2-PDI and ONO-PDI in the solid powder state were recorded using a HR800 labRAM confocal Raman spectrometer, operating at 20 mW laser power using a Peltier cooled (-74° C) CCD detector. Raman spectra were recorded using a He-Ne laser source having an excitation wavelength of 632.8 nm with an acquisition time of 5 s using a 50x objective.

1.7 X-ray Photoelectron Spectroscopic (XPS) Analysis

X-ray photoelectron spectroscopic (XPS) measurements of NO_2-PDI and ONO-PDI solid samples were performed using an ESCA Plus spectrometer (Omicron Nanotechnology Ltd, Germany). Mg Kα (1253.6 eV) was used as the X-ray source operating at 100 watts. General scans and core-level spectra were acquired with1 eV and 50 eV pass energy respectively. Spectral Background (Shirely) de-convolution was done by using CasaXPS software[8].

1.8 Photoirradiation Experiments

The photoirradiation experiments were performed on nitrogen-purged acetonitrile and toluene solutions of NO_2-PDI using 532 nm-10 mW diode laser. The photoirradiation experiment with 532 nm laser was continuously monitored using UV-vis absorption and fluorescence emission measurements.

1.9 Electron Paramagnetic Resonance (EPR) Experiments

Continuous-wave EPR (CW-EPR) measurements with X-band (8.75-9.65 GHz) were carried using JEOL JES-FA200 ESR spectrometer at room temperature and liquid nitrogen (77 K) temperatures. Samples (5 mM conc.) were prepared by loading the solutions of NO_2-PDI in 5 mm o.d. (4 mm i.d.) quartz tubes, subjecting them to nitrogen purging cycles and was sealed later using a rubber septum. Samples were photoexcited inside the EPR cavity with a USHIO Optical Modulex-XENON lamp-ES-UXL 500 (input current = 20 amperes). The acquisition of the EPR samples were carried out with a modulation frequency (100 KHz, width = 0.1 mT), phase (0 degree), sweep time (30.0 s) and time constants of 0.1 and 0.03 s.

Section 2: Syntheses and Characterization

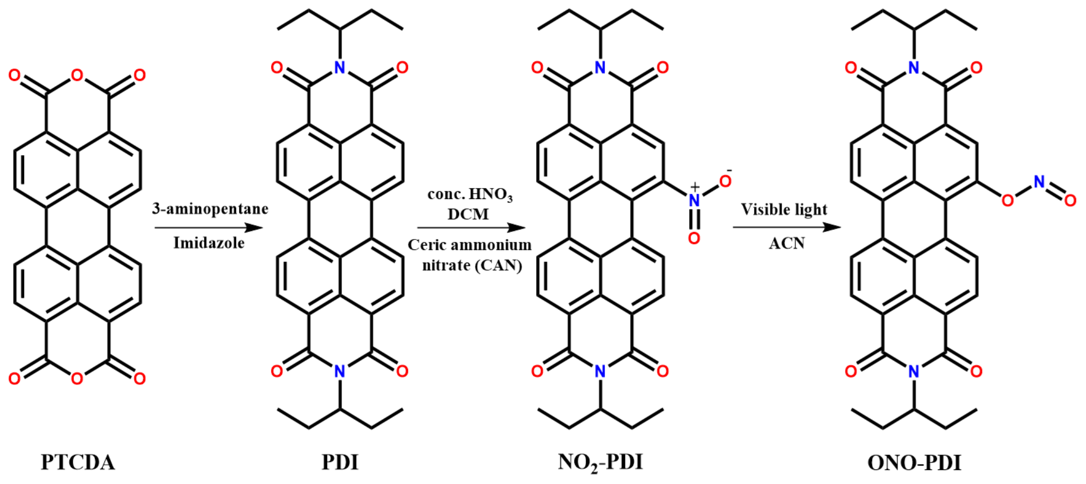

Scheme S1: Showing the synthesis of PDI, NO_2-PDI and ONO-PDI.

2.1 Synthesis of PDI

The compound synthesized according to the literature procedure[9]. 5 g PTCDA was accurately weighed out in a 250 ml round bottom flask. Then about 125 g of Imidazole was added and mixed well. The temperature was raised from room temperature to 140° C. When imidazole melts entirely and forms a homogeneous solution with PTCDA, 2.5 equivalents (3.7 g) of 3-amino pentane were added slowly under the N_2 atmosphere. This reflux was continued for about 6 hr. After the completion of the reaction, washed with a mixture of 1N HCl and Ethanol (40:60). The precipitated product was filtered and purified using column chromatography using DCM: PET ether to afford dark red coloured solid (5.75 g, 85% yield).

M.p. > 300 °C.

^{1}H NMR (500 MHz, $CDCl_3$, ppm) δ = 8.66 (d, J = 8.0 Hz, 4H), 8.60 (d, J = 8.1 Hz, 4H), 5.11 – 5.03 (m, 2H), 2.34 – 2.17 (m, 4H), 1.95 (m, 4H), 0.93 (t,12H).

^{13}C NMR (500 MHz, $CDCl_3$, ppm) δ = 134.7, 131.7, 129.8, 126.7, 123.7, 123.2, 57.9, 25.2, 11.6.

HRMS (APCI) m/z calculated for $C_{34}H_{31}N_2O_4$ [(M+H)$^+$]: 531.2283; found 531.2271.

IR (KBr): cm^{-1} = 2966, 2934, 2876, 1699, 1659, 1595, 1578, 1456, 1434, 1406, 1339, 1252, 1198, 1090, 852, 809, 746.

2.2 Synthesis of NO_2-PDI

The compound was synthesized according to the literature procedure[10]. To a solution of PDI (900 mg, 1.6 mmol) in CH_2Cl_2 (100 mL), conc. HNO_3 (0.1 M, 3.0 mL) and cerium (IV) ammonium nitrate (CAN) (1.2 g, 2.2 mmol) was added and the mixture was stirred at room temperature for 2 hours under N_2. The mixture was neutralized with 10% KOH and was extracted using CH_2Cl_2. The crude product was purified by a silica gel column chromatography, using CH_2Cl_2 as eluent to afford shiny red coloured solid (829 mg, 95% yield).

M.p. > 300 °C.

^{1}H NMR (500 MHz, $CDCl_3$, ppm) δ = 8.74 (d, *J* = 7.6 Hz, 1H), 8.62–8.69 (m, 4H), 8.55 (d, *J* = 8.0 Hz, 1H), 8.18 (d, *J* = 7.6 Hz, 1H), 4.93-4.99 (m, 2H), 2.12-2.21 (m, 4H), 1.83-1.89 (m, 4H), 0.84 (t, *J* = 10 Hz, 12H).

^{13}C NMR (500 MHz, $CDCl_3$, ppm) δ = 147.70, 135.50, 132.95, 129.53, 129.38, 129.14, 127.95, 127.53, 126.68, 126.47, 124.53, 124.07, 58.24, 57.95, 24.99, 24.91, 11.33, 11.31.

HRMS (APCI) m/z calculated for $C_{34}H_{30}O_6N_3$ [(M+H)$^+$]; 576.2129; found 576.2112.

IR (KBr): cm^{-1} = 2963, 2947, 2878, 1705, 1659, 1597, 1535, 1458, 1404, 1335, 1250, 1204, 1088, 856, 810, 748.

2.3 Synthesis of ONO-PDI

To a 20 mL quartz test tube equipped with a magnetic stir bar was charged with NO_2-PDI (50 mg, 0.087 mmol) in acetonitrile (10 mL) as the solvent. The test tube was backfilled with argon and sealed, then irradiated with a 532 nm 10mW-diode laser for 2 hours at room temperature. The crude product was purified by silica gel column chromatography using EtOAc as eluent to afford yellow coloured solid (3.8 mg, 7.6% yield).

M.p. = 288 °C.

^{1}H NMR (500MHz, $CDCl_3$, 300 K), δ = 8.87-8.90 (t, *J* = 10 Hz, 2H), 8.63-8.64 (t, *J* = 5 Hz, 3H), 8.36 (s, 1H), 7.94 (s, 1H), 4.92-4.99 (m, 2H), 2.10-2.21 (m, 4H), 1.83-1.92 (m, 4H), 0.84-0.87 (t, *J* = 10 Hz, 12H).

HRMS (APCI) m/z calculated for $C_{34}H_{30}O_6N_3$ [$(M+H)^+$]: 576.2129; found 576.2120.

IR (KBr): cm^{-1} = 2966, 2937, 2876, 1707, 1664, 1462, 1390, 1360, 1335, 1304, 1240, 1184, 1014, 901, 841, 800, 743.

^{1}H NMR spectra of both NO_2-PDI and ONO-PDI showed the presence of 7 aromatic deshielded protons and 22 aliphatic shielded protons. The observance of the ^{1}H NMR singlet peak (single proton) shifting from 8.64 ppm in NO_2-PDI to 8.36 ppm in ONO-PDI confirms the rearrangement of the nitro (-NO_2) group to nitrito (-ONO) group thereby altering the associated electronic effects (Figure S4-S6, ^{13}C NMR spectrum of ONO-PDI could not be obtained due to the very less stability of the compound in solution state for long scan times)[11]. CHN elemental microanalysis was employed to measure the percentage elemental composition of the isomers NO_2-PDI and ONO-PDI. Interestingly, both the isomers exhibited almost similar elemental composition percentages which were correlated with the theoretical/calculated data available. NO_2-PDI displayed an error of 0.36% from the calculated data, while ONO-PDI displayed a marginal error of 0.17% from the calculated data (hexane used for solvent correction, Table S2)[12]. Molecular mass analysis of the red coloured isomer NO_2-PDI and the yellow coloured isomer ONO-PDI was accomplished using high resolution mass spectrometry-atmospheric pressure chemical ionization (HRMS-APCI). Both the compounds demonstrated the same molecular ion peak. NO_2-PDI molecular ion peak was observed at m/z=576.2116 $(M+H)^+$ (Figure S7a) while ONO-PDI molecular ion peak was observed at m/z=576.2120 $(M+H)^+$ (Figure S7b). The indication of the same molecular ion mass peak in HRMS spectra verifies the rearrangement of nitro to nitrito functionality in the course of photoisomerization.

Section 3: Tables

Table S1: Selected publications showing the evolution of nitrite linkage isomers reported over the years.

Contributed by	Year	Molecule Exhibiting Nitro to Nitrito Linkage Isomerization	Method/Technique of Characterization
Genth and co-worker<br>*Am. J. Sci.* **1857**, *24*, 86.	**1857**	$[Co(NH_3)_5NO_2]^{+2}$	Chemical complexation studies
Werner<br>*Ber.* **1907**, *40*, 765-788.	**1907**	$O_2NC_nH_{2n+1}$	Chemical complexation studies
Basolo and co-workers<br>*J. Am. Chem. Soc.* **1954**, *76*, 5920.	**1954**	cis-$[Coen_2NO_2H_2O]^{+2}$	Reaction mechanism studies
Hammaker and co-worker<br>*Inorg. Chem.* **1962**, *1*, 1-5	**1962**	$[(NH3)_5RhONO]^{+2}$	UV-vis absorption and IR studies
Nordin and co-worker<br>*Inorg. Chem.* **1979**, *18*, 1869-1874.	**1979**	$[Co(NH_3)_5NO_2]Cl_2$	X-ray crystallographic studies
Adamo and co-worker<br>*J. Phys. Chem. A* **2001**, *105*, 1086-1092.	**2001**	$[Co(NH_3)_5ONO]^{+2}$	Theoretical analysis using hybrid density functional/Hartree-Fock method
Boldyreva and co-workers<br>*Angew. Chem. Int. Ed.* **2013**, *52*, 9990-9995.	**2013**	$[Co(NH_3)_5(NO_2)]Cl(NO_3)$	Kinematic analysis of photomechanical stress
Andrew and co-workers<br>*Inorg. Chem.* **2017**,*56*,13205-13213.	**2017**	Cytochrome *c'*	Resonance Raman, FTIR and density functional theory
Hariharan and co-workers<br>Current work	**2022**	Nitro-perylenediimide	XPS, FTIR, Raman, ^1H NMR, CHN, HRMS, ultrafast spectroscopy and density functional theory

Table S2: NO_2-PDI and ONO-PDI CHN elemental analysis (corrected with hexane as solvent).

Molecule	C (%)	H (%)	N (%)	Error (%)
NO_2-PDI (calc.)	70.95	5.08	7.30	—
NO_2-PDI (expt.)	71.96	5.35	7.11	0.37
ONO-PDI (expt.)	71.57	5.34	7.13	0.17

Table S3: Oscillator strength for different transitions of NO_2-PDI and ONO-PDI calculated at TD-CAM-B3LYP/ 6-311++G(d,p) level of theory.

Molecule	Excitation		Energy (eV)	Wavelength (nm)	Main Transition Orbital
	State	Oscillator Strength (f)			
**NO_2-PDI**	S_1	0.7612	2.7276	454.56	HOMO → LUMO
	S_2	0.0200	3.7493	330.69	HOMO-1 → LUMO<br>HOMO → LUMO+1
	S_3	0.0562	3.7665	329.18	HOMO → LUMO+1
	S_4	0.0449	3.9172	316.51	HOMO-1 → LUMO
**ONO-PDI**	S_1	0.8073	2.7189	456.01	HOMO → LUMO
	S_2	0.0453	3.2069	386.62	HOMO-11 → LUMO+1<br>HOMO → LUMO+1
	S_3	0.0012	3.7964	326.59	HOMO-1 → LUMO
	S_4	0.0115	3.9044	317.55	HOMO → LUMO+1

Table S4: UV-vis and fluorescence spectroscopic data of NO_2-PDI and ONO-PDI measured in toluene at room temperature.

	**NO_2-PDI**	**ONO-PDI**
$\lambda_{abs}(A_{0-0})$, nm	521	377
$\lambda_{abs}(A_{0-1})$, nm	489	356
$\lambda_{abs}(A_{0-2})$, nm	454	346
λ_{em}, nm	458	459
$\Delta\tilde{\nu}_{Stokes}$, nm	63	82
ϕ_{Fl}, %	<1	<1

Table S5: Lowest-energy triplet states of nitro-perylenediimide (NO_2-PDI) and the respective triplet quenchers used.

Molecule	Lowest Triplet Energy (eV)
Nitro-perylenediimide	1.2
β-Carotene	0.9
Anthracene	1.8
Naphthalene	2.6
Perylene	1.6
Ferrocene	1.7
Fullerene-C_{60}	1.5
1-Bromopyrene	2.1

Table S6: Spin-orbit coupling values between four bright states (S_1, S_2, S_3, S_4) and low lying triplet states at NO2-PDI minimum evaluated at TD-CAM-B3LYP/6-311++G(d,p) level of theory using PySOC package implemented in Gaussian 16.

States	SOC values (cm^{-1})
S_1/T_2	0.65
S_1/T_3	4.50
S_1/T_4	1.17
S_2/T_8	2.35
S_3/T_8	2.58
S_3/T_9	1.90
S_3/T_{10}	1.16
S_4/T_{10}	0.61

Table S7: Ground state (S_0) optimized geometrical parameters of NO_2-PDI, ONO-PDI and TS at CAM-B3LYP/6-311++G(d,p) level of theory.

Geometrical parameters	Molecule		
	NO_2-PDI	ONO-PDI	TS
(NO_2/ONO)-aromatic torsion angle (degrees)	59.87	-127.57	52.35
Coretwist (degrees)	16.07	-9.49	2.82
Energy (kcal mol^{-1})	-1210010.62	-1210010.50	-1209933.01
Dipole moment (debye)	4.19	1.47	1.61

Table S8: Natural Bond Orbital (NBO) interactions supporting the proposed intramolecular mechanism of photoisomerization of NO_2-PDI to ONO-PDI evaluated at CAM-B3LYP/6-311 ++G(d,p) level of theory.

Donor(i)	Type	Acceptor(j)	Type	E(2) kcal mol^{-1}	E(j)-E(i) a.u.	F(i,j) a.u.
N31-O32	σ	C8-H36	σ*	13.09	1.41	0.122
N31-O32	π	C8-H36	σ*	8.40	0.76	0.072
N31-O33	σ	C8-H36	σ*	1.04	1.38	0.034
O32	LP (1)	C8-H36	σ*	2.31	1.12	0.046
O33	LP (1)	C8-H36	σ*	1.50	1.15	0.037
O33	LP (2)	C8-H36	σ*	5.75	0.78	0.061
O33	LP (3)	C8-H36	σ*	15.34	0.54	0.095

Table S9: Theoretical Bond Order of selected bonds in the proposed 6-membered TS calculated from the Wiberg Bond Indices evaluated in Multiwfn 3.8.

Bond	Calculated Bond Order
C8-O42	0.60
C8-H46	0.25
C17-N41	0.69
C17-H46	0.43
N41-O42	0.82
N41-O43	1.44

Table S10: Completely optimized geometrical parameters along with the absolute energies of ground-state (S_0), relaxed singlet excited-state (S_1^{CR}) and singlet Franck-Condon state (S_1^{FC}) of NO_2-PDI computed at CAM-B3LYP/6-311++G(d,p) level of theory.

Geometrical parameters	Molecule		
	S_0	S_1^{CR}	S_1^{FC}
nitro-aromatic torsion angle (degrees)	59.9	33.4	59.9
Coretwist (degrees)	16.1	13.3	16.1
Energy (kcal mol^{-1})	-1210010.62	-1209953.94	-1209947.72
Dipole moment (debye)	4.1	4.3	-

Table S11: Calculated dipole moment values of the transition state in vacuum, polar acetonitrile and non-polar toluene solvent models at CAM-B3LYP/6-311++G(d,p)) level of theory.

Optimized Structure	Dipole Moment (Debye)
Transition state (vacuum)	1.6
Transition state (acetonitrile)	1.9
Transition state (toluene)	1.7

Section D: Figures

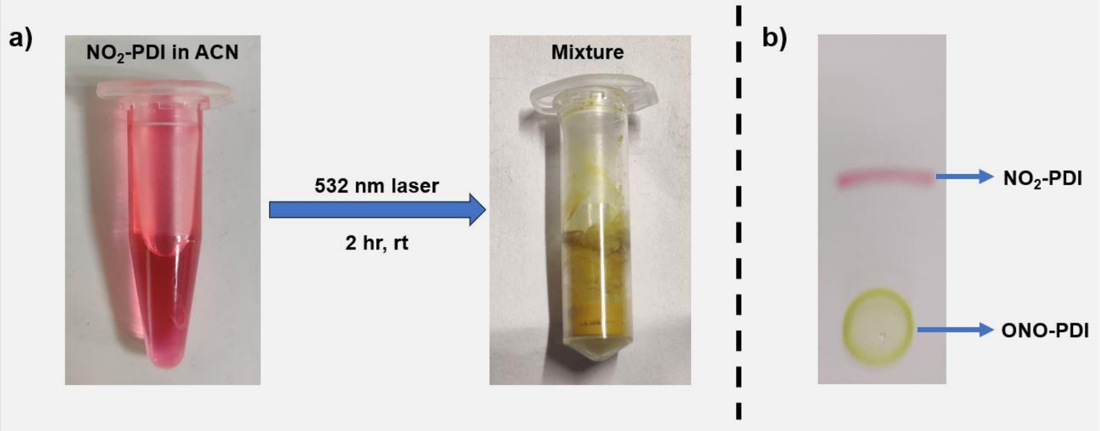

Figure S1: a) Transformation of NO_2-PDI to yellow coloured ONO-PDI under visible light irradiation for 2 hrs in acetonitrile. b) TLC showing red coloured NO_2-PDI and yellow coloured ONO-PDI (DCM:EtOAc=2:1).

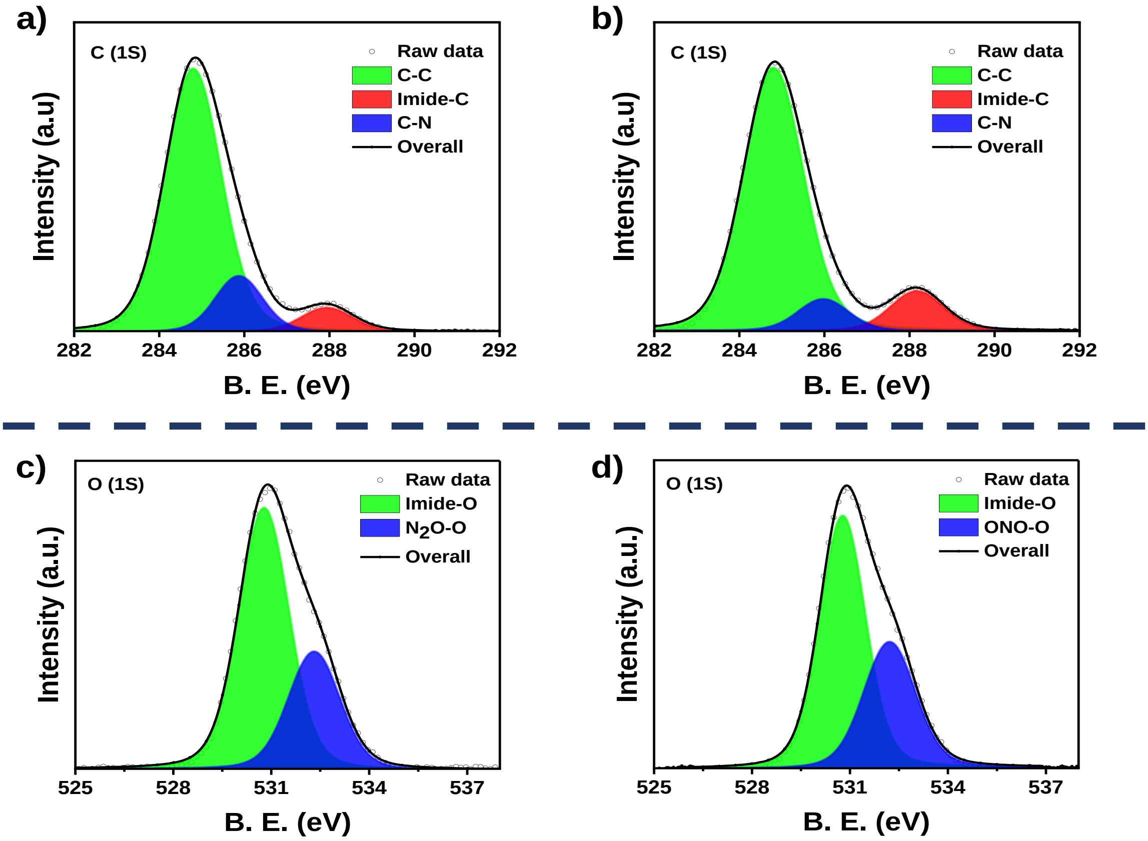

Figure S2: (Top) Core level C (1S) XPS spectrum of a) NO_2-PDI and b) ONO-PDI. (Bottom) Core level O (1S) spectrum of c) NO_2-PDI and d) ONO-PDI.

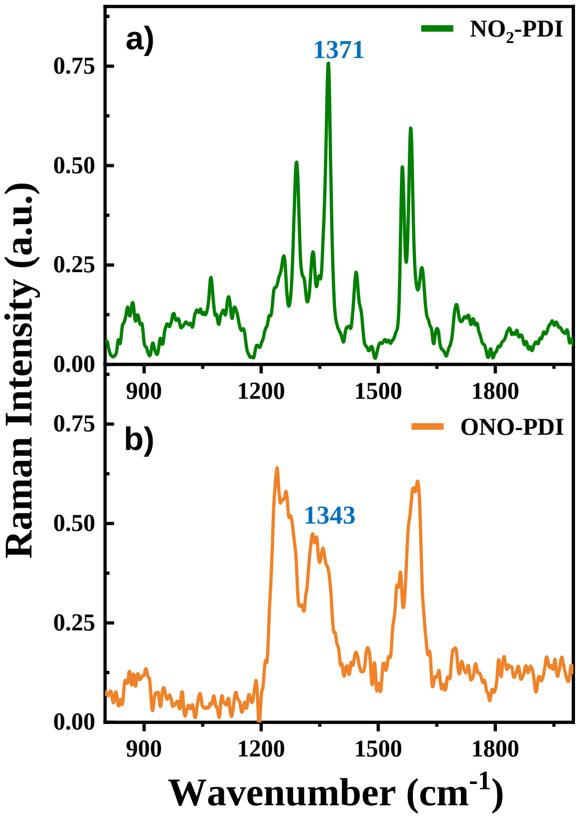

Figure S3: Raman spectra of a) NO_2-PDI and b) ONO-PDI measured in the powder state after photoexcitation at 632.8 nm.

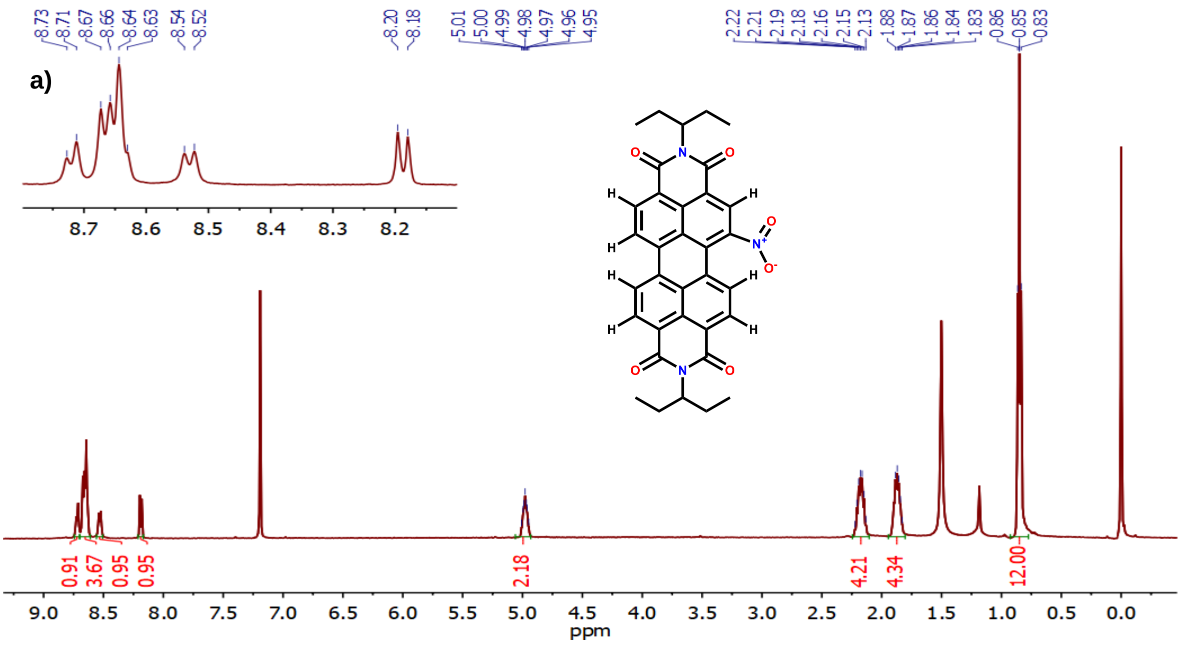

Figure S4: ^1H-NMR spectrum of NO_2-PDI in $CDCl_3$. Inset a) shows the splitting of the core aromatic protons.

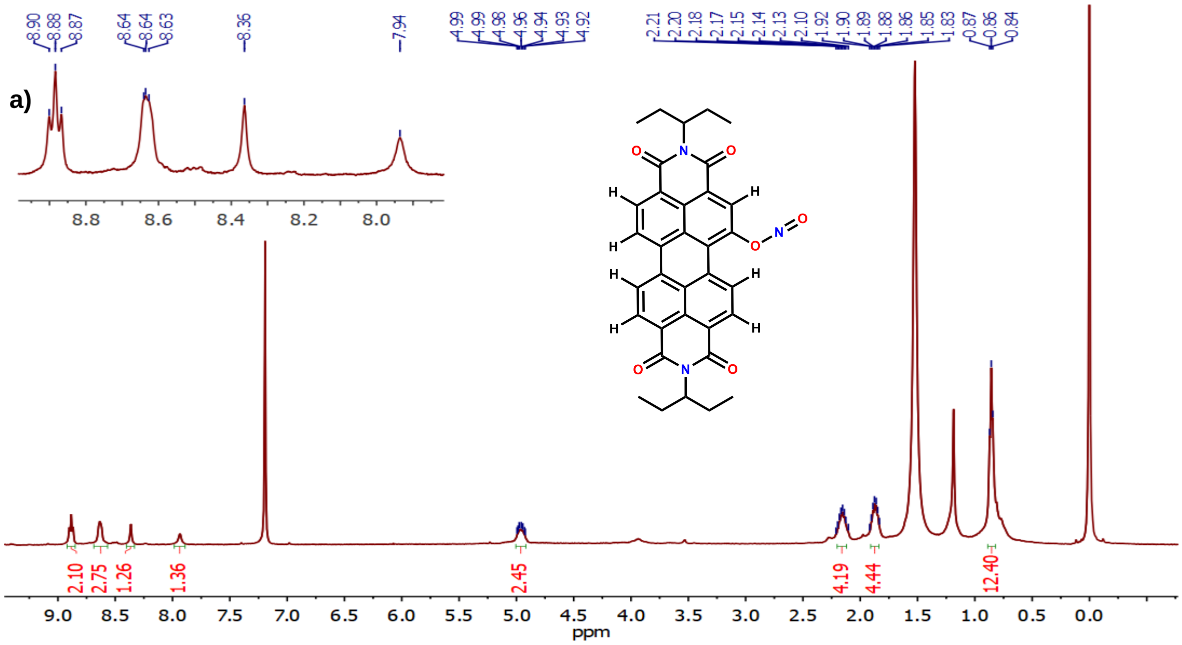

Figure S5: ^{1}H-NMR spectrum of ONO-PDI in $CDCl_3$. Inset a) shows the splitting of the core aromatic protons.

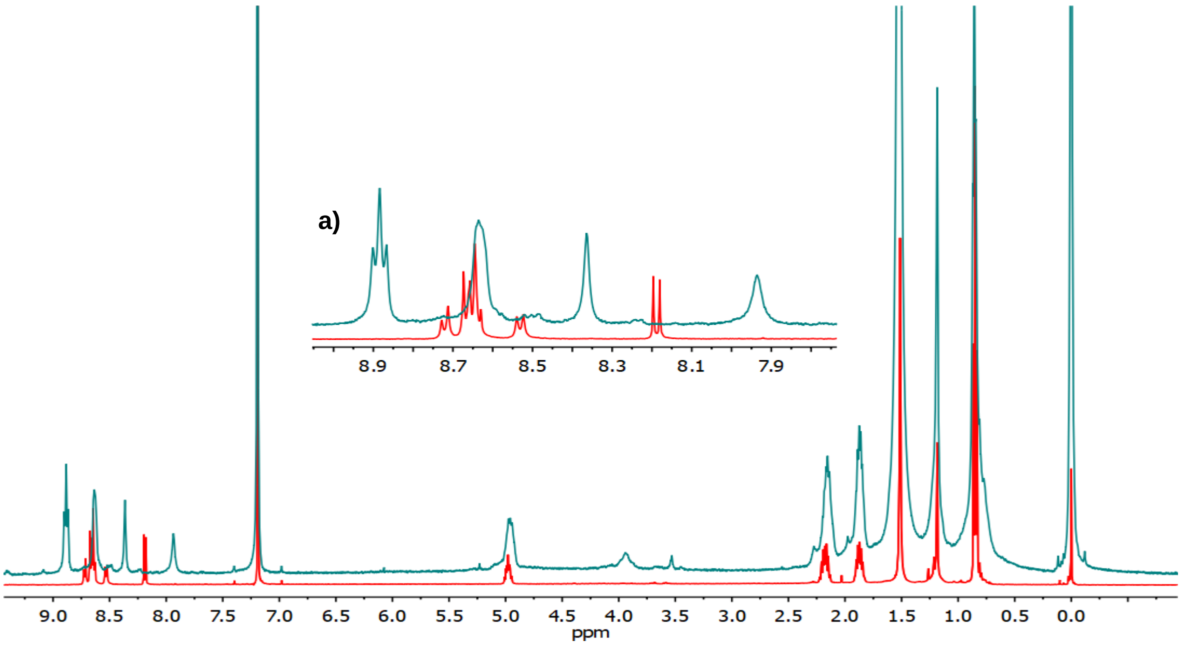

Figure S6: Superimposed ^{1}H-NMR spectra of NO_2-PDI (red spectrum) and ONO-PDI (blue spectrum) in $CDCl_3$. Inset a) shows the splitting of the core aromatic protons.

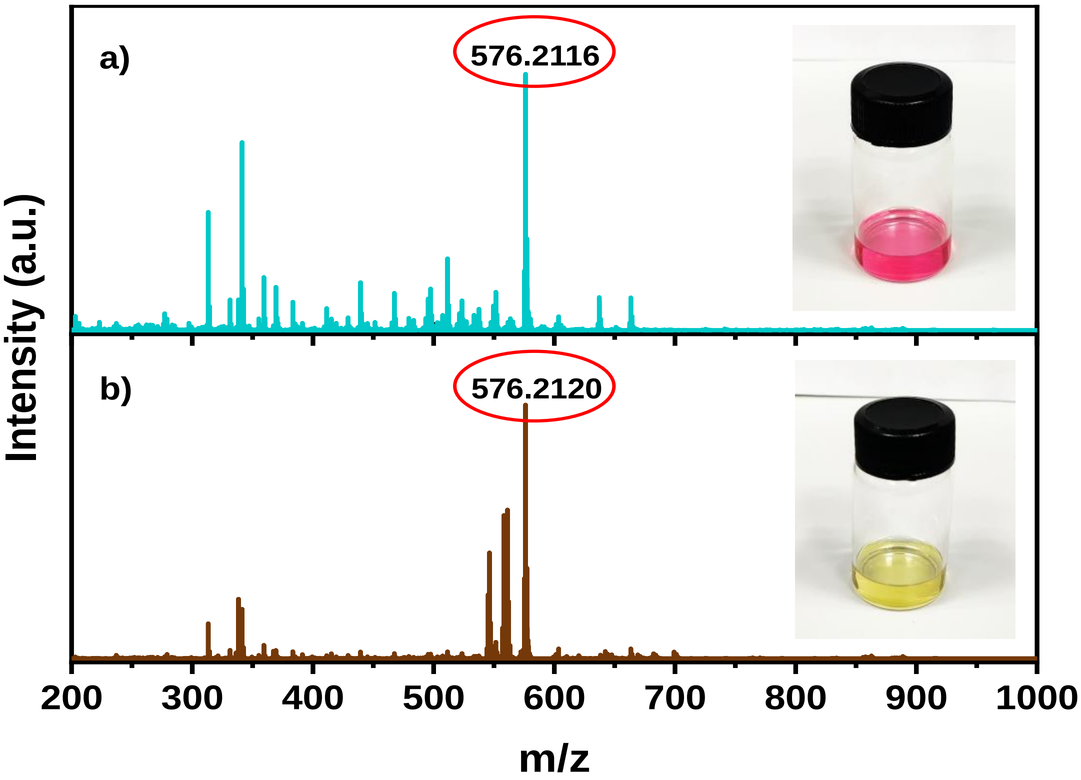

Figure S7: HRMS (APCI) spectra of a) NO_2-PDI and b) ONO-PDI in $CHCl_3$.

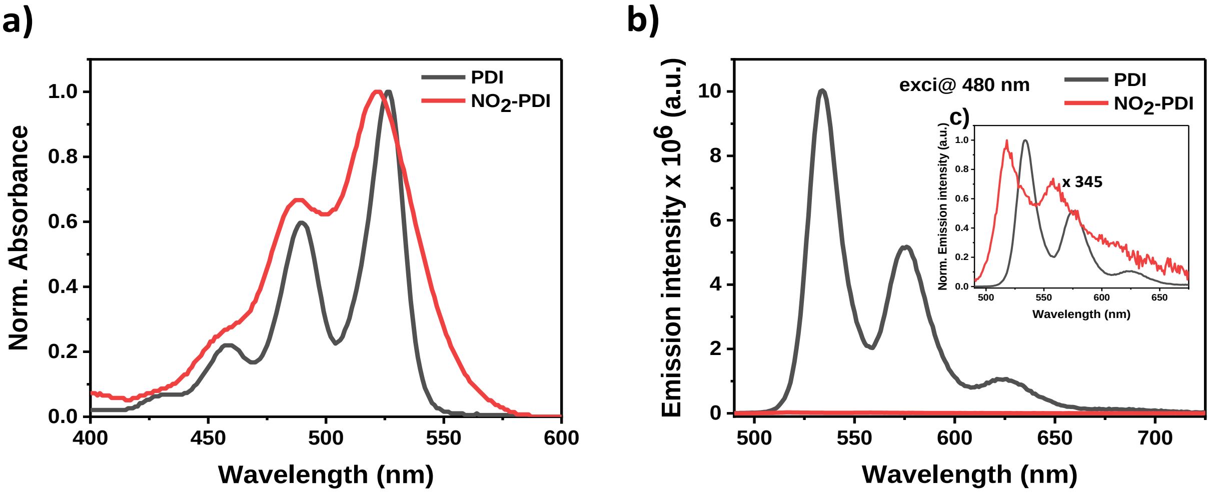

Figure S8: a) Normalized absorption spectra of PDI (black line) and NO_2-PDI (red line), b) Emission spectra of PDI (black line) and NO_2-PDI (red line) in $CHCl_3$ at room temperature. Zoomed inset c) Showing the normalized emission of PDI (black line) and NO_2-PDI x 345 units (red line).

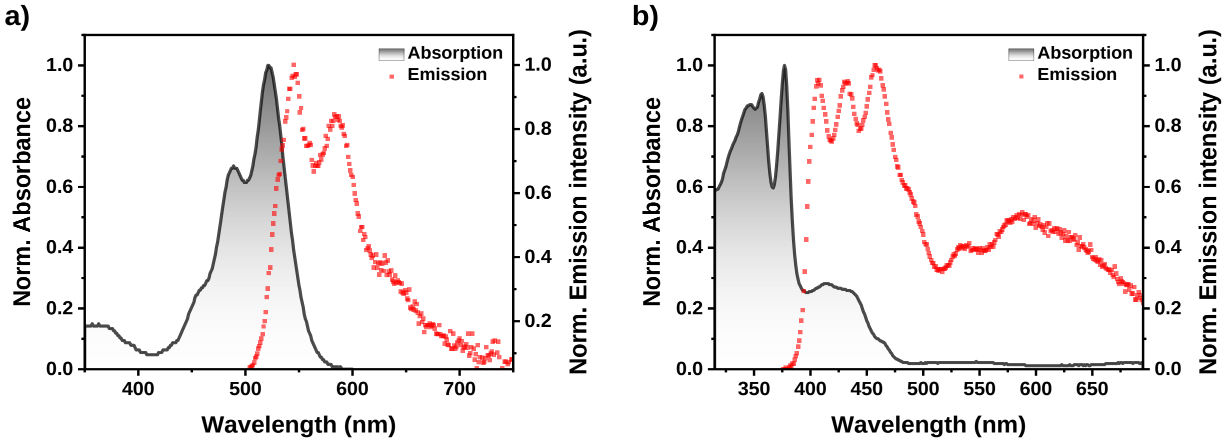

Figure S9: Normalized absorption (solid black line) and emission spectra (dotted red line) of a) NO_2-PDI and b) ONO-PDI in $CHCl_3$ at room temperature.

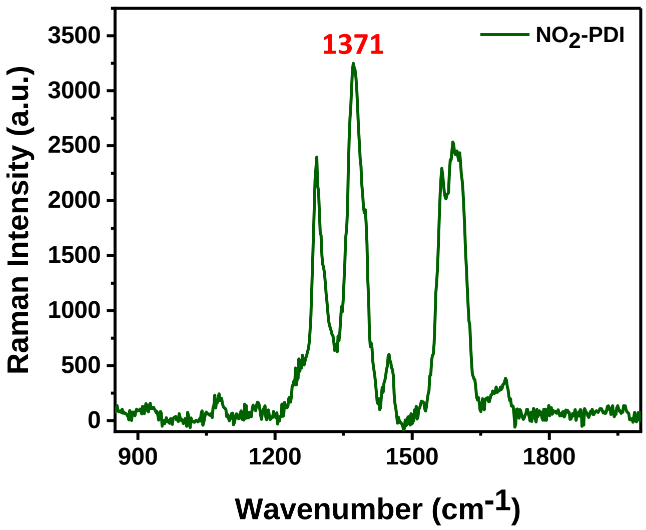

Figure S10: Resonant Raman spectrum of NO_2-PDI measured in the powder state upon excitation at 488 nm with an acquisition time of 5 s using a 50x objective.

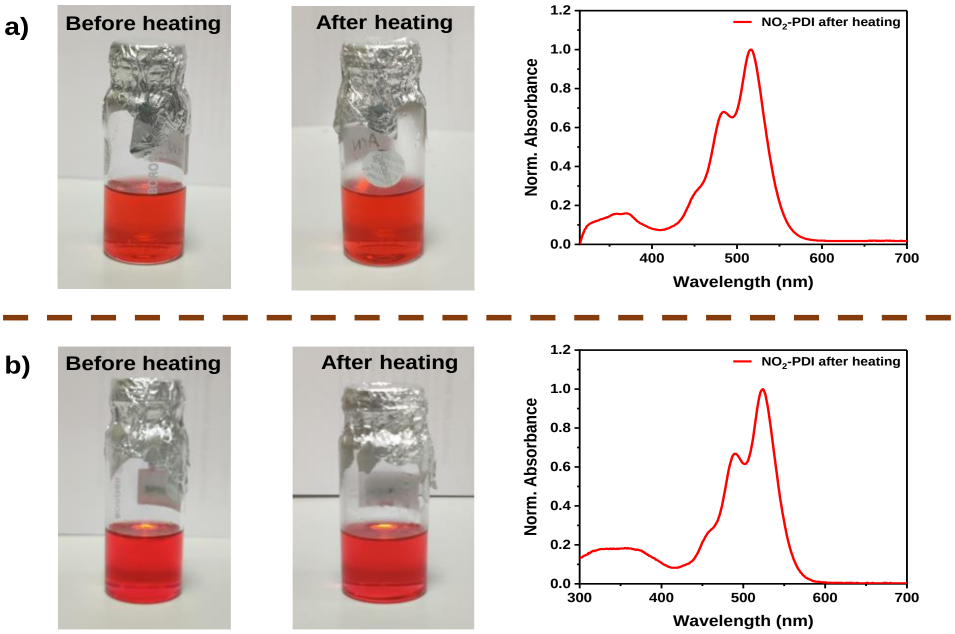

Figure S11: NO_2-PDI solutions before and after heating along with the UV-vis absorption spectrum in a) acetonitrile and b) toluene.

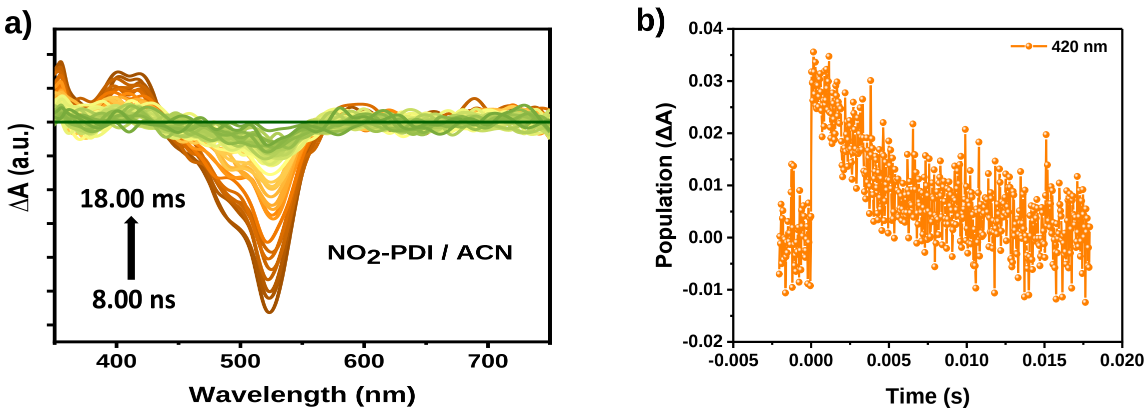

Figure S12: a) nsTA (λ_{ex} = 532 nm) spectra of NO_2-PDI in acetonitrile. b) Decay trace showing excited-state absorption at 420 nm obtained from nsTA measurements of NO_2-PDI in acetonitrile.

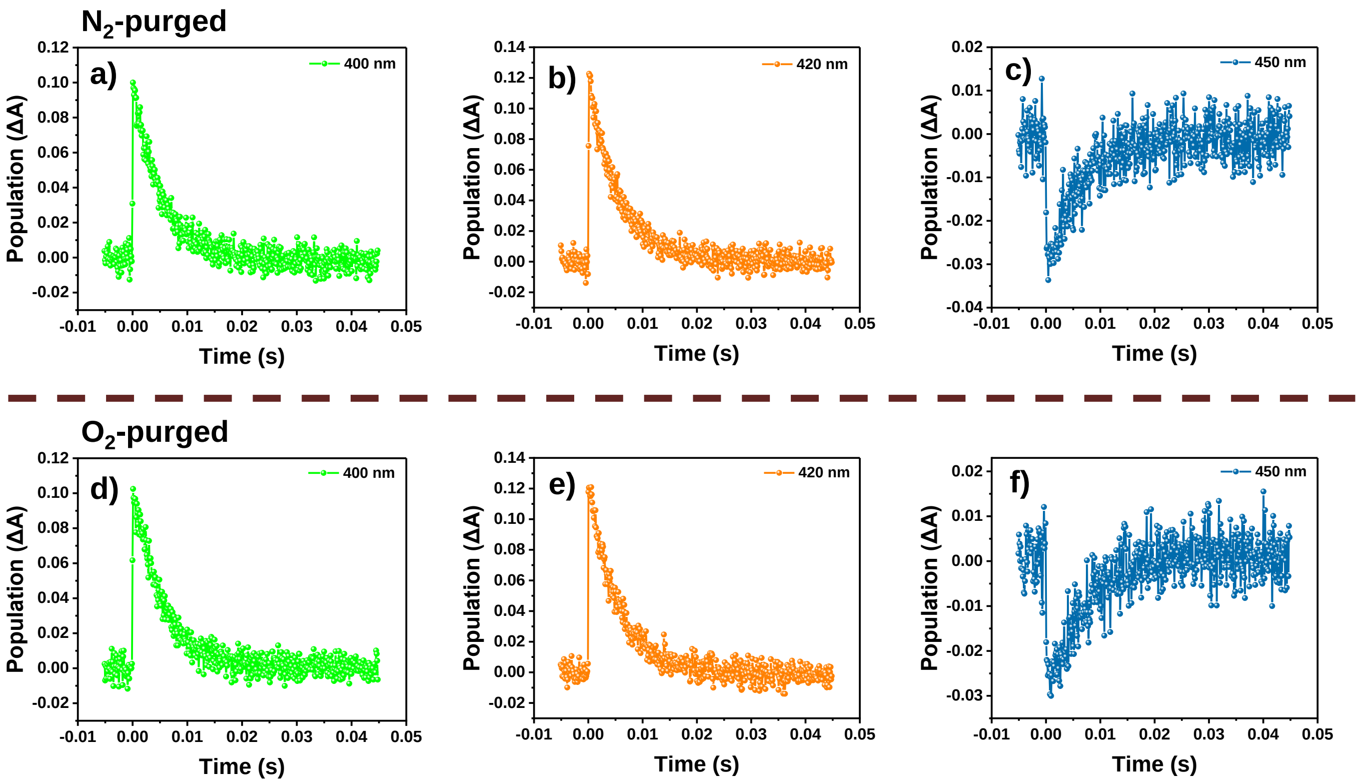

Figure S13: (Top) nsTA features of NO_2-PDI in nitrogen-purged toluene solution at a) 400 nm, b) 420 nm and c) 450 nm. (Bottom) Triplet quenching studies of NO_2-PDI in oxygen-purged toluene solution at d) 400 nm, e) 420 nm and f) 450 nm.

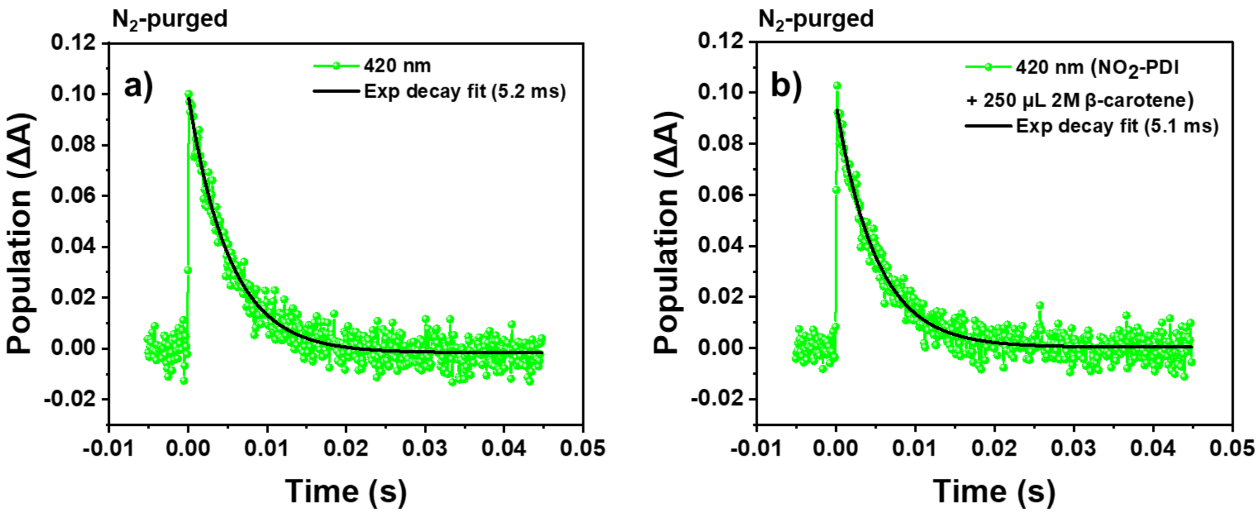

Figure S14: a) nsTA features of NO_2-PDI in nitrogen-purged toluene solution at 420 nm. b) Triplet-triplet energy transfer studies of NO_2-PDI using 250 μL 2M β-carotene at 420 nm.

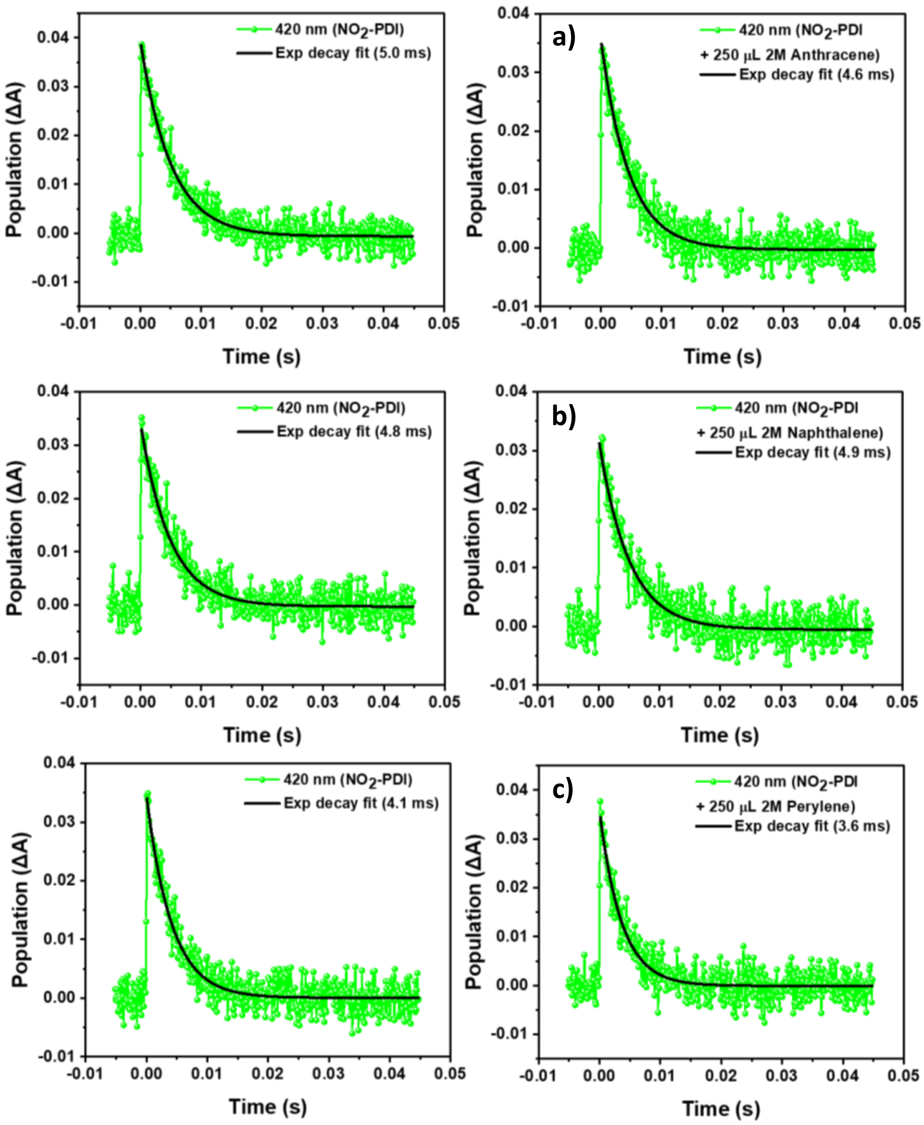

Figure S15: (Left) nsTA features of NO_2-PDI in nitrogen-purged toluene solution at 420 nm. (Right) Triplet-triplet energy transfer studies of NO_2-PDI probed at 420 nm using 250 μL 2M a) Anthracene, b) Naphthalene and c) Perylene.

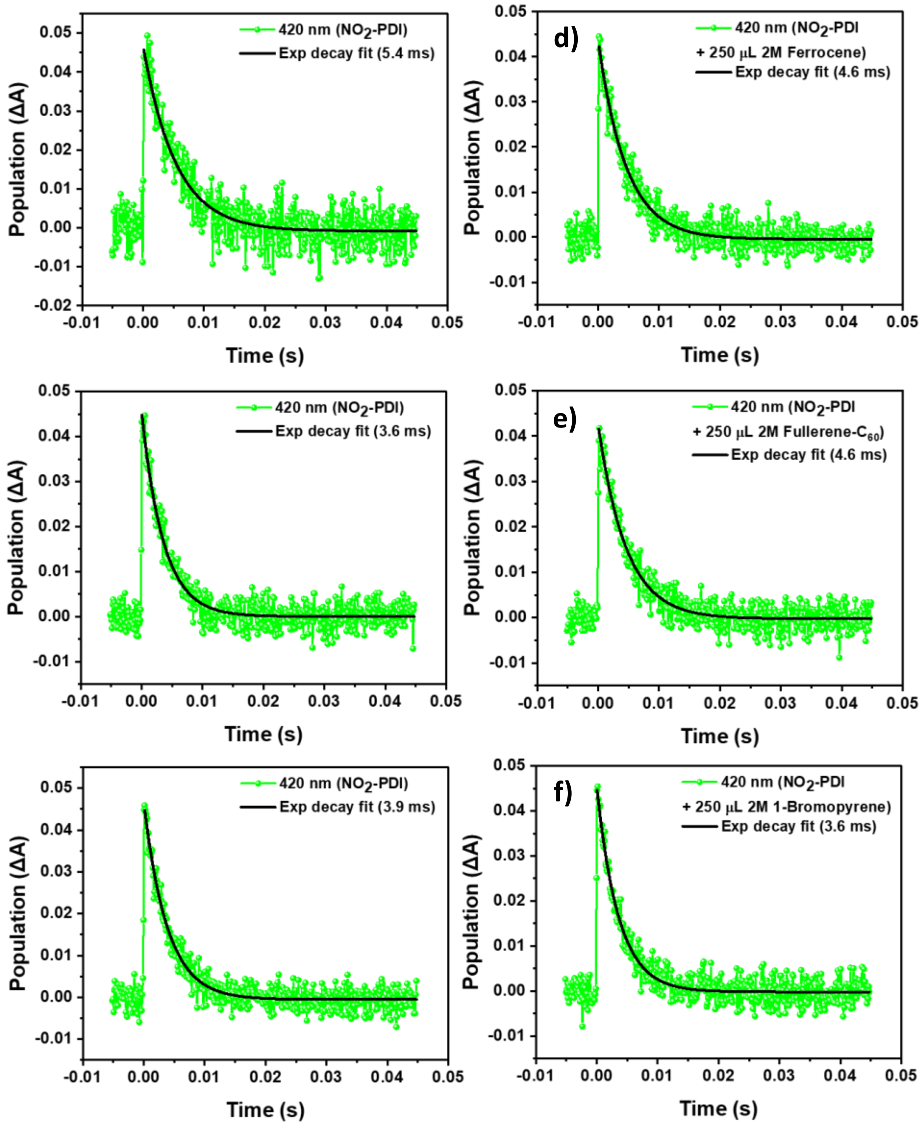

Figure S16: (Left) nsTA features of NO_2-PDI in nitrogen-purged toluene solution at 420 nm. (Right) Triplet-triplet energy transfer studies of NO_2-PDI probed at 420 nm using 250 μL 2M d) Ferrocene, e) Fullerene-C_{60} and f) 1-Bromopyrene.

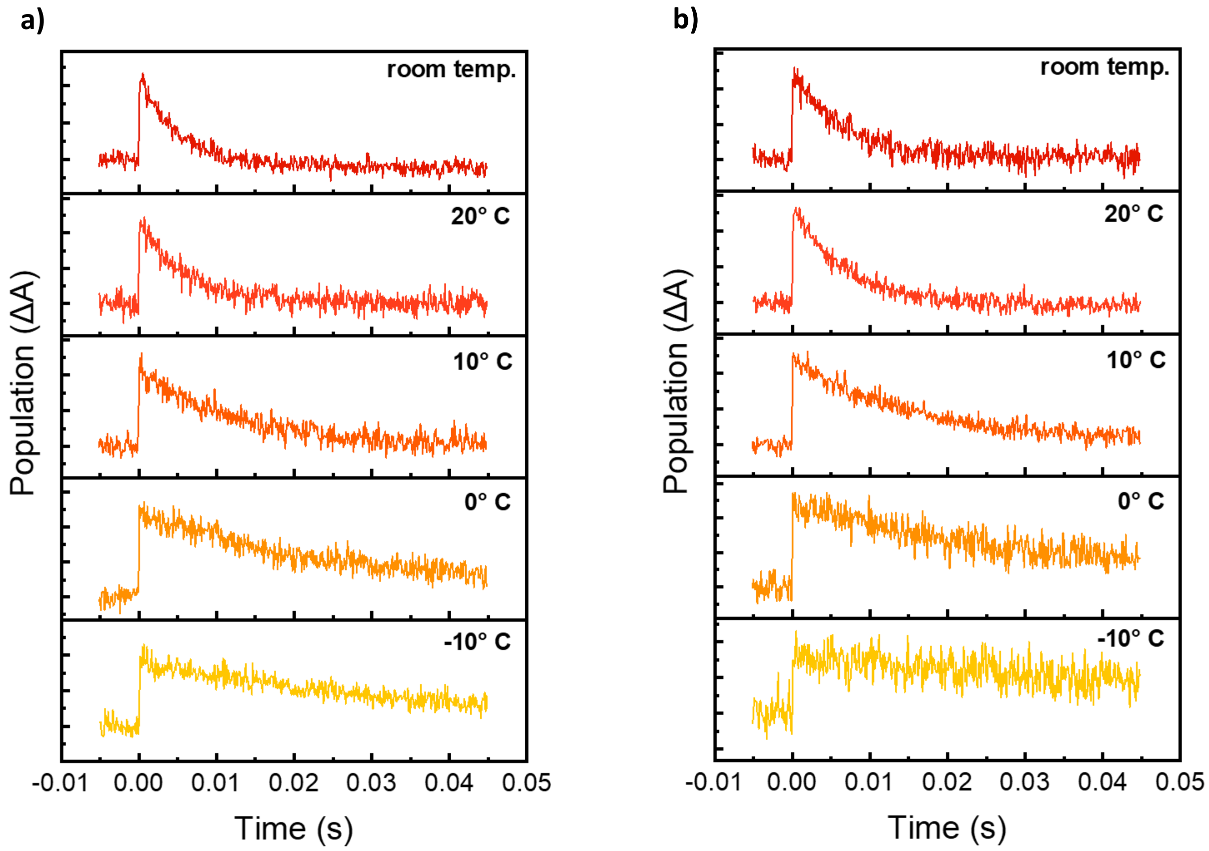

Figure S17: Temperature-dependent nsTA spectra of NO_2-PDI in a) toluene and b) acetonitrile. N. B.- At lower temperatures the lifetime of the NO_2-PDI transient feature increases and the decay of this transient species is not complete. The most probable reason for the increase in the lifetime of the transient species is the rigidification of the molecule at lower temperatures, thereby reducing the total degrees of freedom and suppression of the deactivation channels of NO_2-PDI.

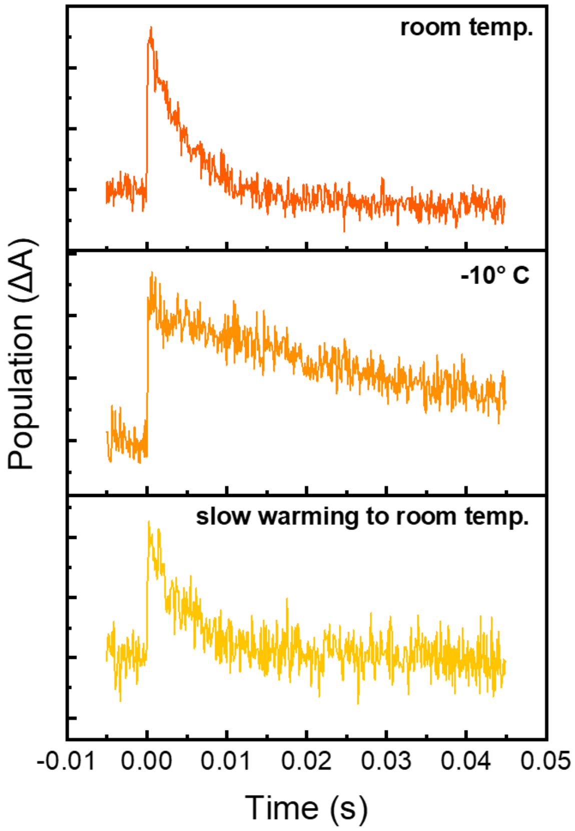

Figure S18: Temperature-dependent nsTA spectra of NO_2-PDI in toluene, showing the decay traces at room temperature, -10° C and slow warming to room temperature conditions. N. B.- The persistence of the long-lived transient feature in toluene at room temperature, -10° C and slow warming to room temperature conditions clearly demonstrates the excited-state photophysical transformation of the NO_2-PDI molecule and rules out the possibility of the observed long-lived excited-state to be a photochemical species.

a)

b)

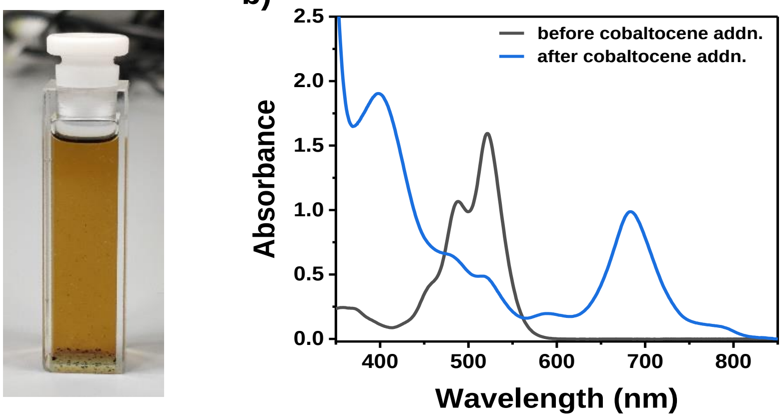

Figure S19: a) NO_2-PDI radical anion solution after cobaltocene addition. b) UV-vis absorption spectra of NO_2-PDI before and after addition of cobaltocene.

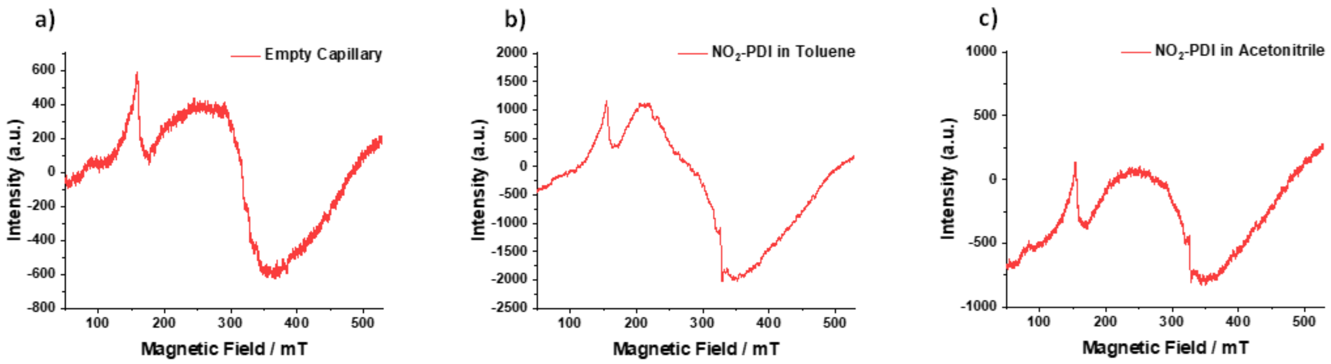

Figure S20: Electron paramagnetic resonance (EPR) spectrum of a) Empty sample capillary, b) NO_2-PDI in toluene solvent and c) NO_2-PDI in acetonitrile solvent recorded at room temperature under continuous light irradiation. N. B.- At room temperature no prominent peak was observed in the NO_2-PDI EPR spectrum in toluene and acetonitrile, and only background noise from the sample capillary was observed.

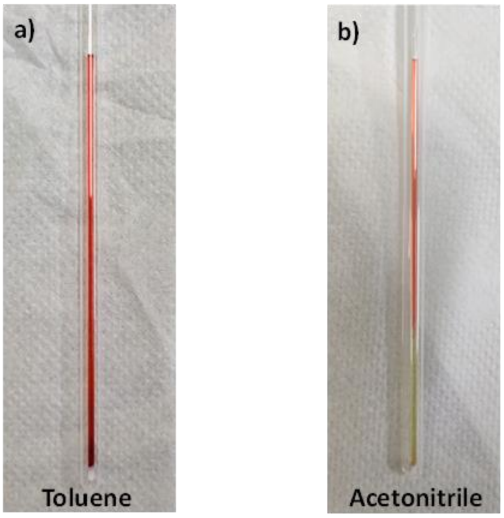

Figure S21: NO_2-PDI sample after EPR measurements under continuous light irradiation conditions at room temperature in a) toluene and b) acetonitrile solvents. N. B.- Red colour of NO_2-PDI sample was intact in toluene after EPR experiment, whereas red coloured NO_2-PDI transformed to yellow coloured ONO-PDI after EPR experiment in acetonitrile.

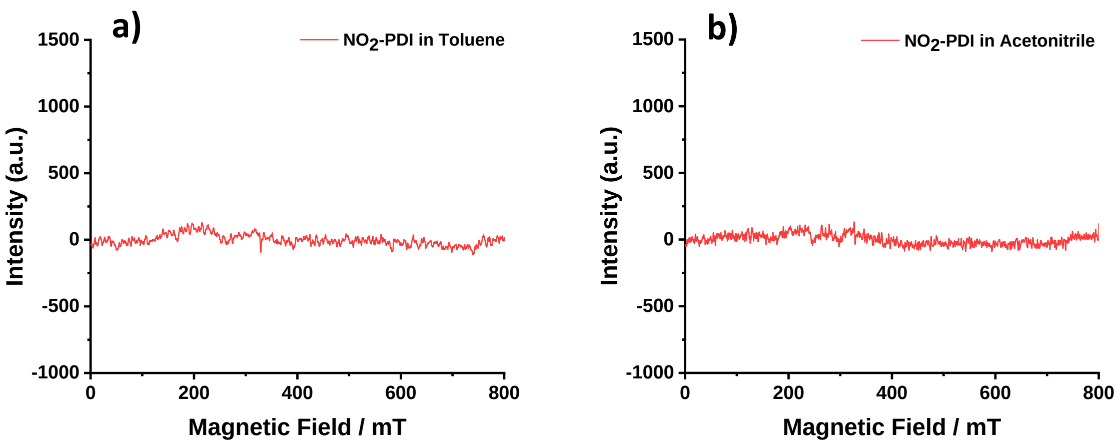

Figure S22: Electron paramagnetic resonance (EPR) spectrum of a) NO_2-PDI in toluene and b) NO_2-PDI in acetonitrile solvents recorded at liquid nitrogen temperature (77 K).

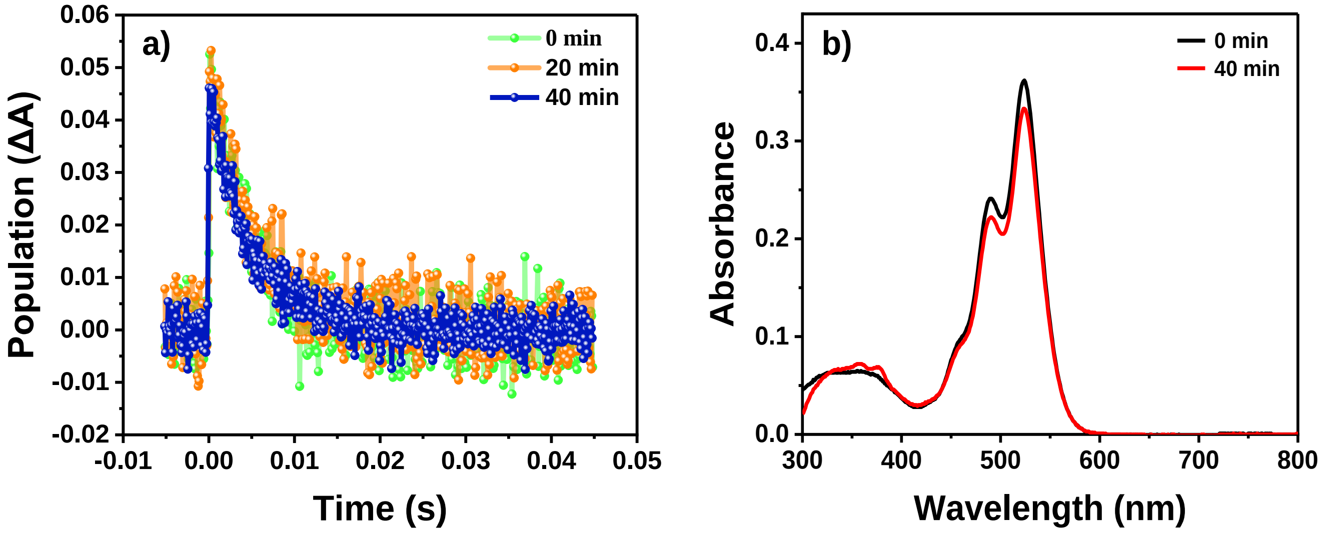

Figure S23: a) Single wavelength (420 nm) nsTA decay kinetics of NO_2-PDI in toluene at 0 min, 20 min and 40 min. b) UV-vis absorption spectra of NO_2-PDI at 0min and 40 min in toluene.

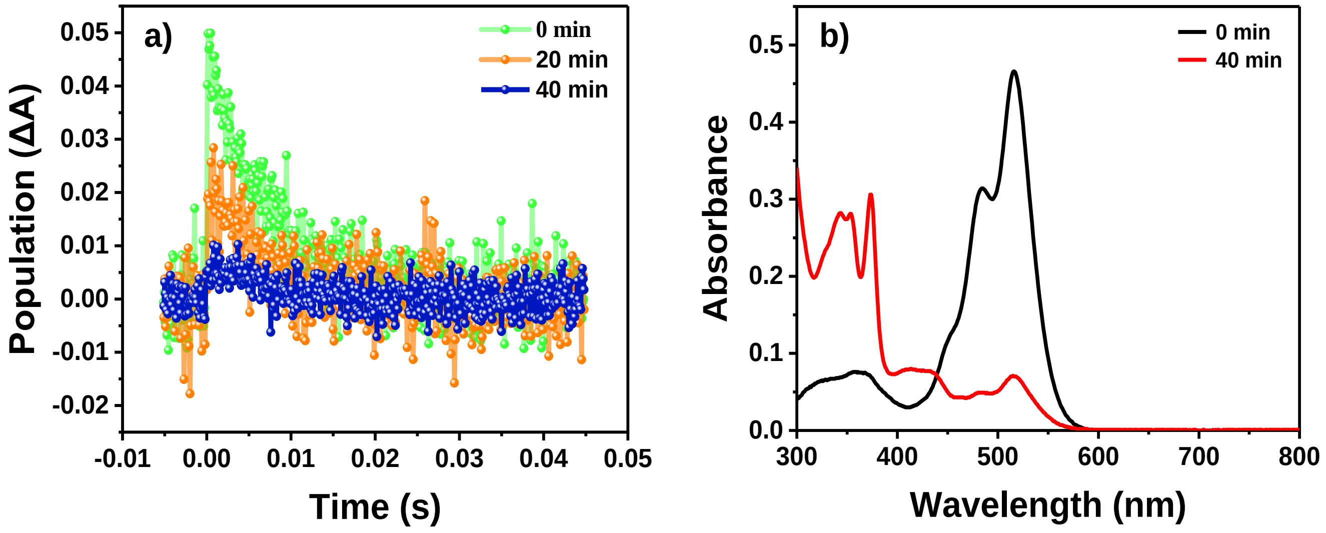

Figure S24: a) Single wavelength (420 nm) nsTA decay kinetics of NO_2-PDI in acetonitrile at 0 min, 20 min and 40 min. b) UV-vis absorption spectra of NO_2-PDI at 0min and 40 min in acetonitrile.

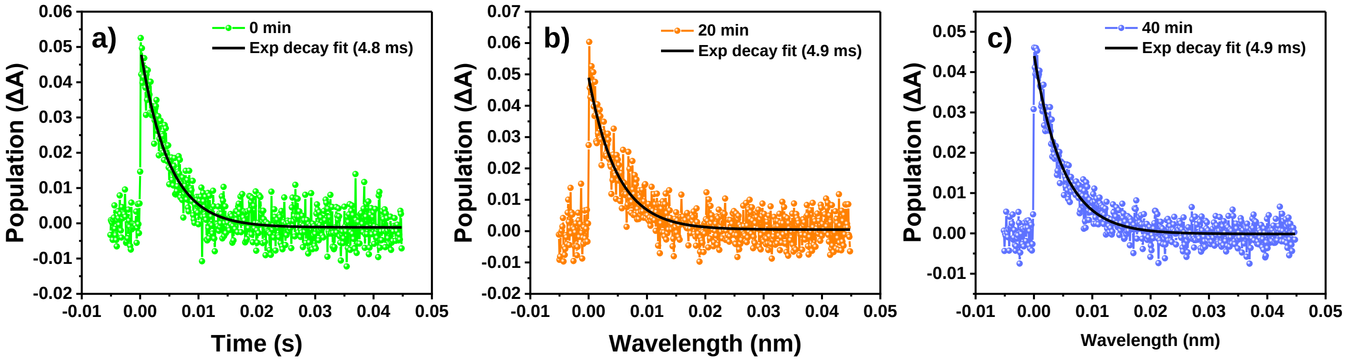

Figure S25: Exponentially fitted decay lifetime of the excited-state absorption of NO_2-PDI in toluene at a) 0 min, b) 20 min and c) 40 min obtained through the single wavelength (nsTA) decay kinetics experiment.

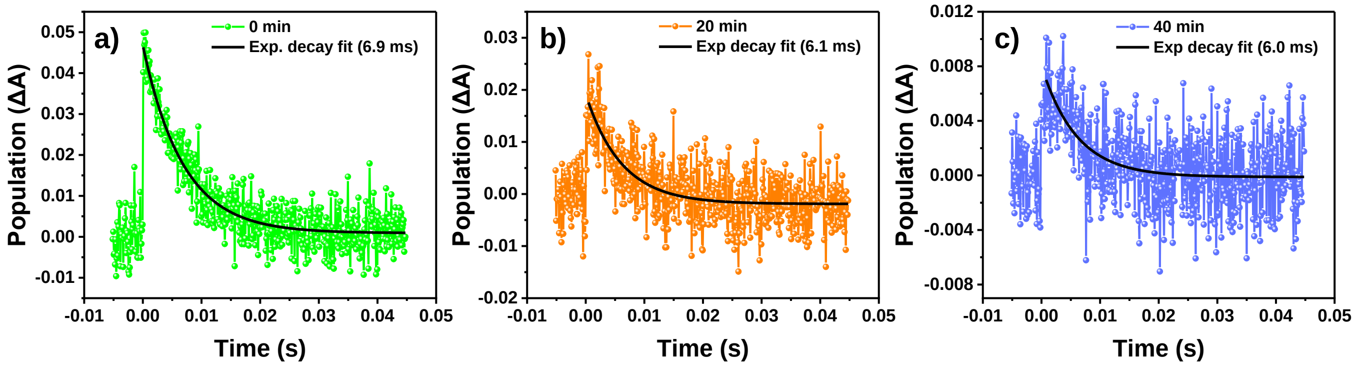

Figure S26: Exponentially fitted decay lifetime of the excited-state absorption of NO_2-PDI in acetonitrile at a) 0 min, b) 20 min and c) 40 min obtained through the single wavelength (nsTA) decay kinetics experiment.

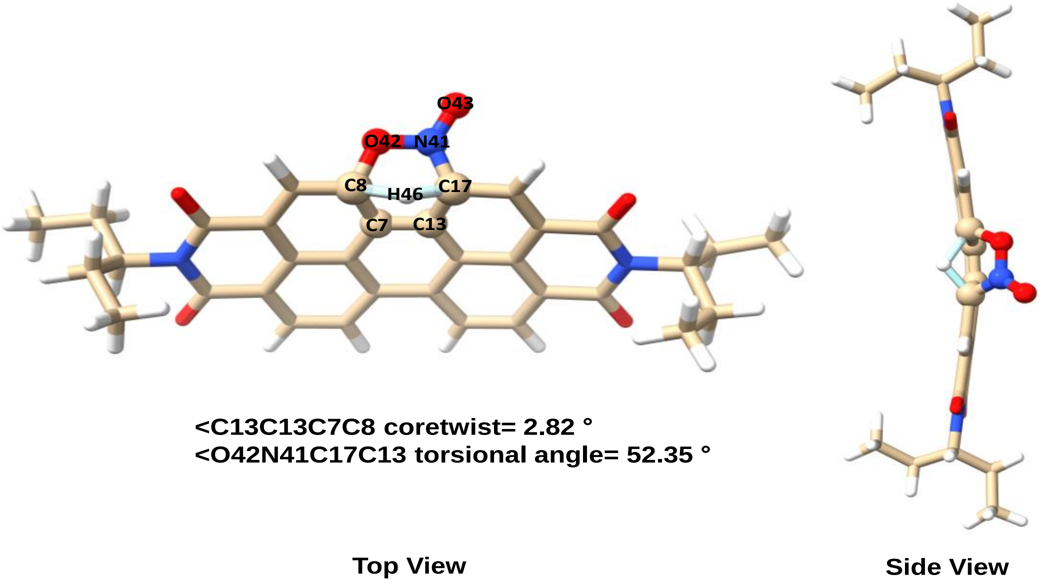

Figure S27: Optimized 6-membered transition state geometry at CAM-B3LYP/6-311++G(d,p) level of theory.

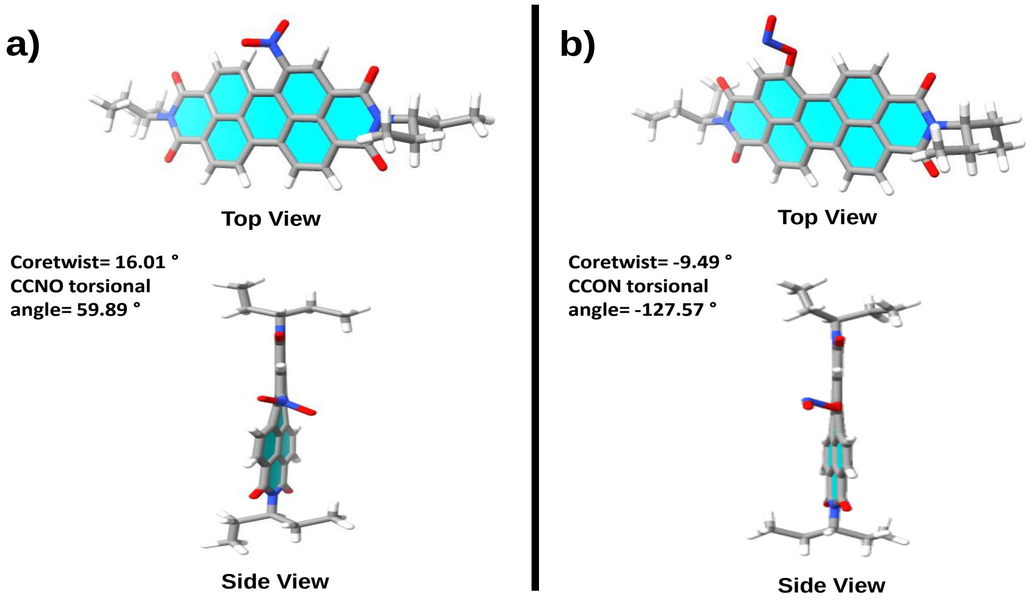

Figure S28: a) NO_2-PDI and b) ONO-PDI optimized geometries at CAM-B3LYP/6-311++G(d,p) level of theory.

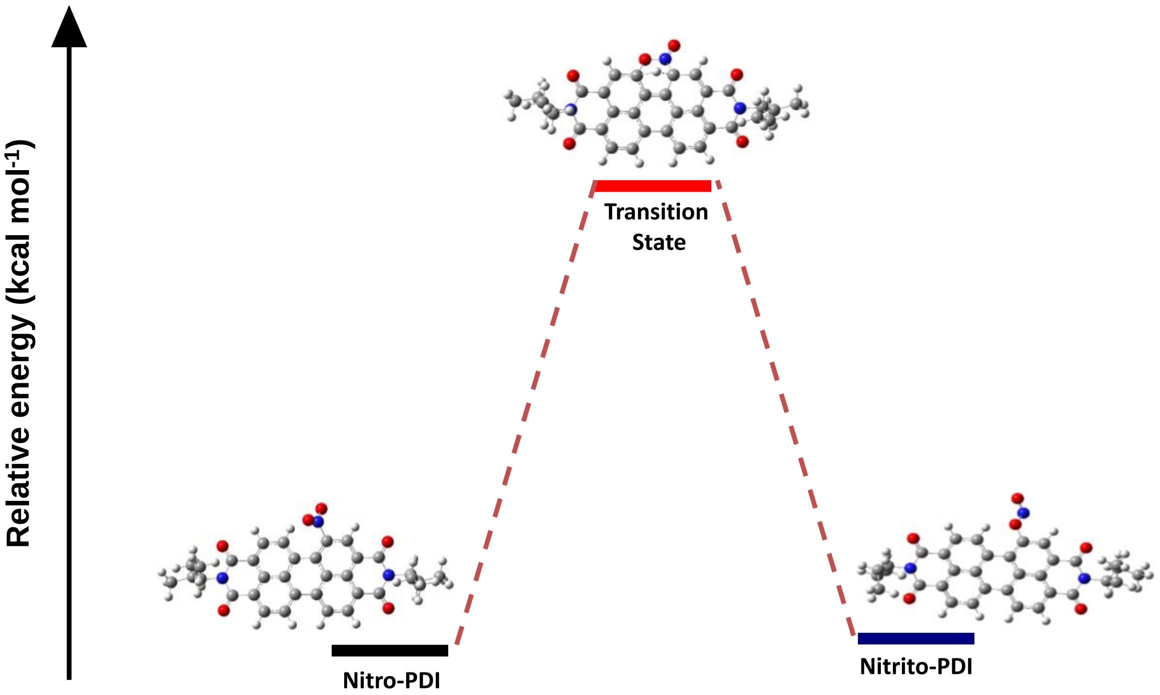

Figure S29: Relative energy ordering of NO_2-PDI, Transition state and ONO-PDI. Energies calculated with the respective optimized geometries at CAM-B3LYP/6-311++G(d,p) level of theory.

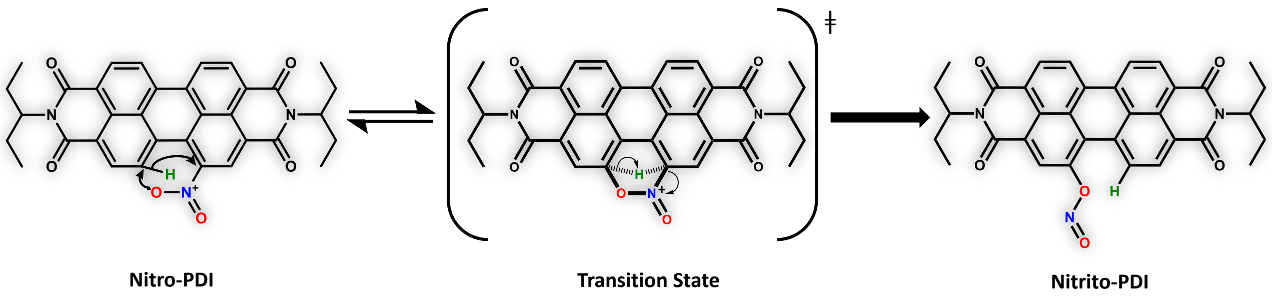

Figure S30: Plausible intramolecular rearrangement mechanism of NO_2-PDI photoisomerization to ONO-PDI.

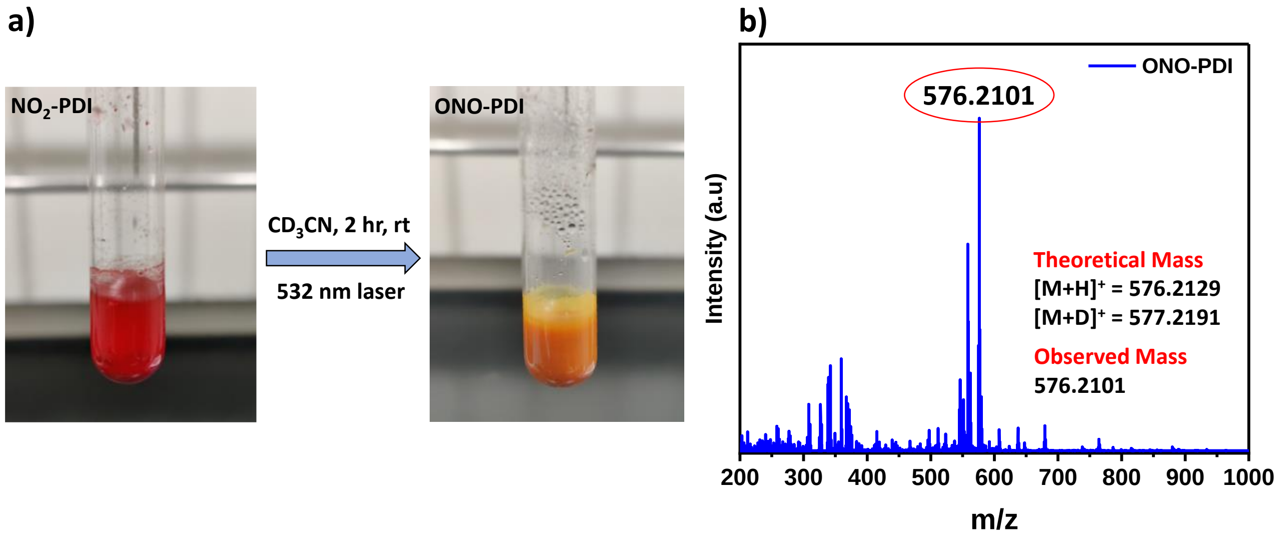

Figure S31: a) Transformation of NO_2-PDI to yellow coloured ONO-PDI under visible light irradiation for 2 hrs in deuterated acetonitrile (CD_3CN). b) HRMS spectrum showing the presence of non-incorporated deuterium product.

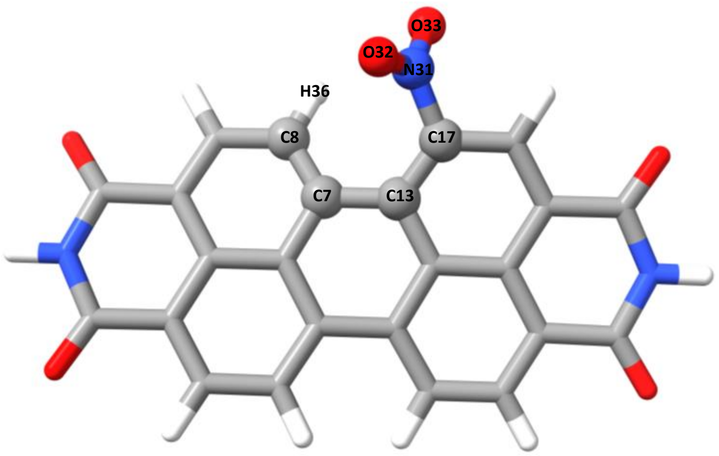

Figure S32: NO_2-PDI structure considered for Natural Bonding Orbital (NBO) calculations (3-aminopentane group is replaced by H atom to reduce computational cost).

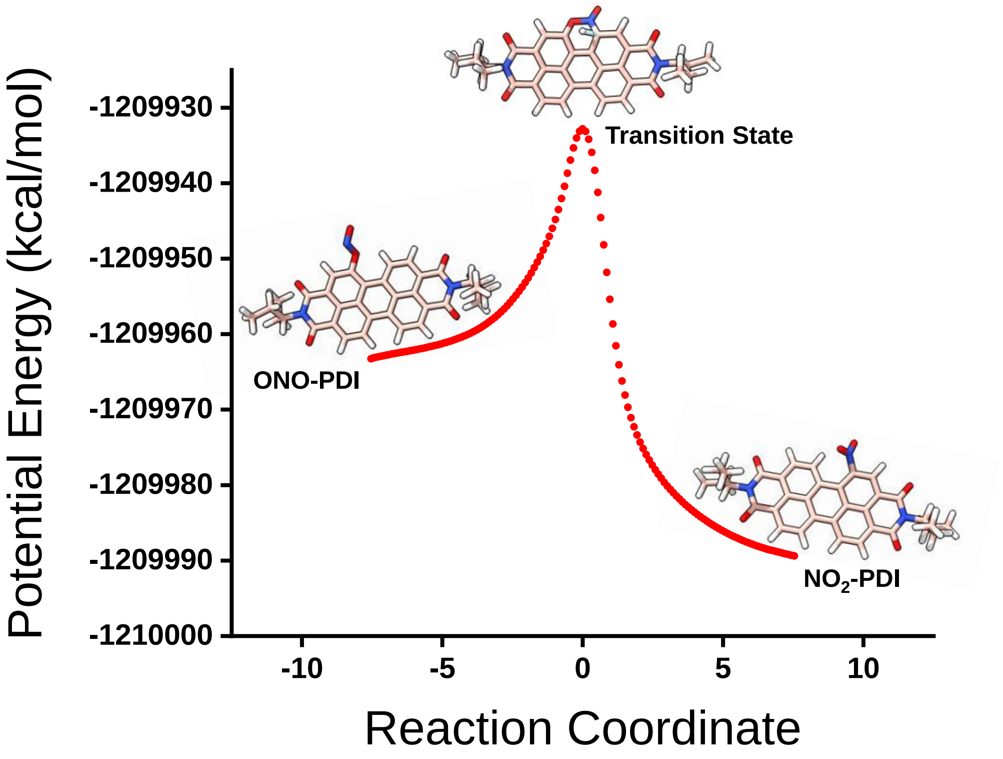

Figure S33: Intrinsic reaction coordinate (IRC) pathway of photoisomerization of NO_2-PDI to ONO-PDI calculated at CAM-B3LYP/6-311++G(d,p) level of theory.

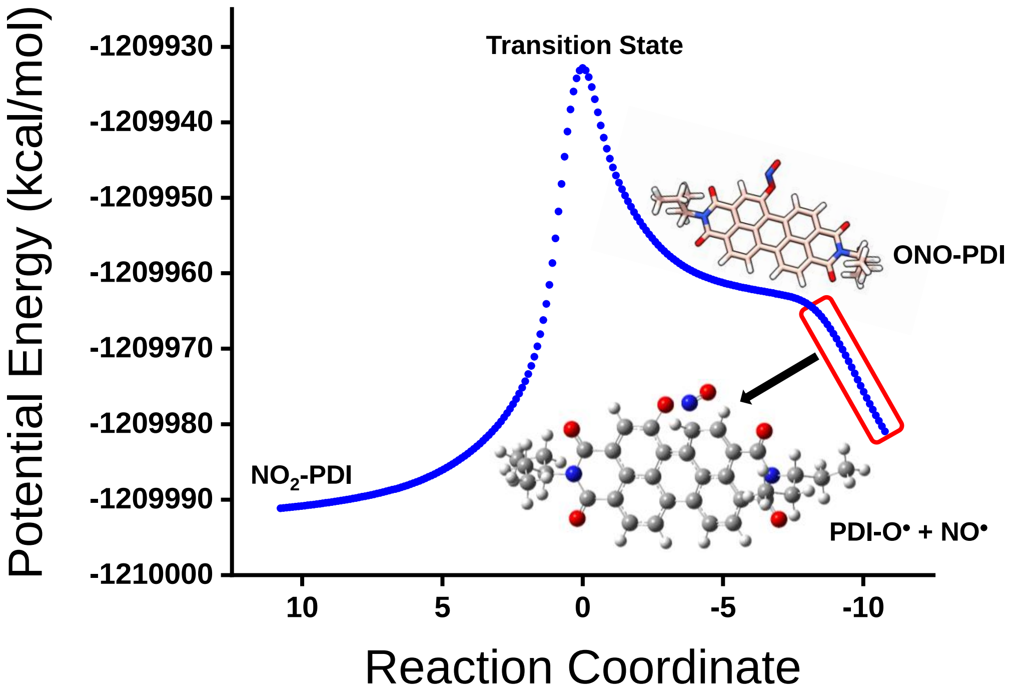

Figure S34: Intrinsic reaction coordinate (IRC) pathway showing the dissociation of ONO-PDI into nitrosyl and aryloxy free radicals below the ONO-PDI minimum computed at CAM-B3LYP/6-311++G(d,p) level of theory.

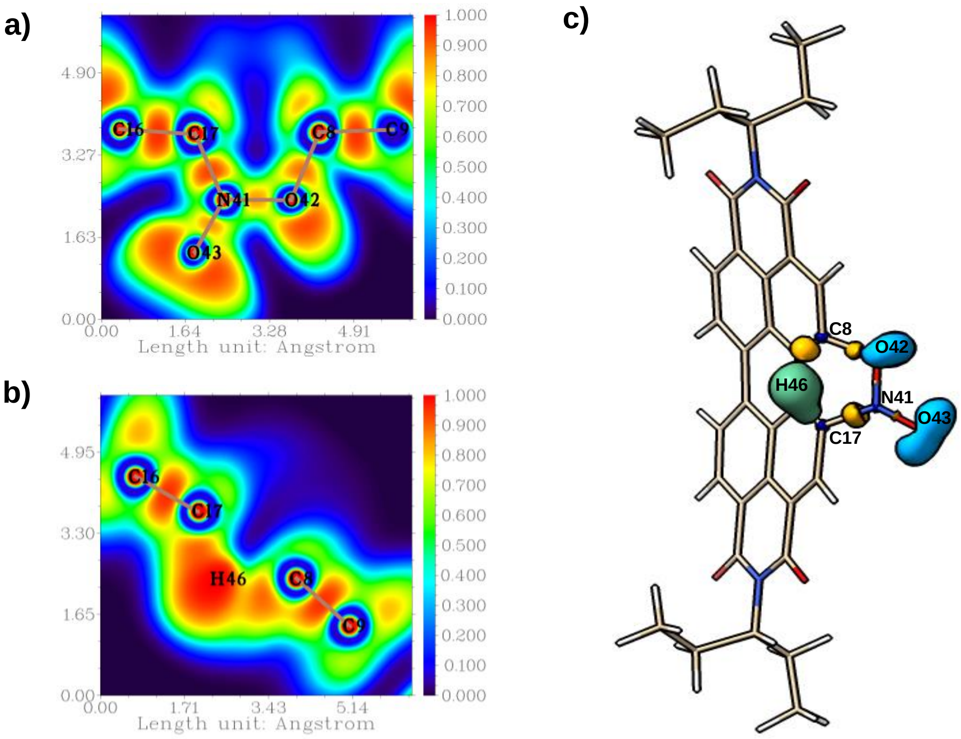

Figure S35: 2D-Electron localization function (ELF) plots of a) TS showing bond formation betweeen O42-C8 atoms (O42N41C17 considered as the plane of reference) and b) TS showing the bond formation between C17-H46 and bond breaking between C8-H46 (C17H46C8 considered as the plane of reference). c) 3D-ELF plot of the TS.

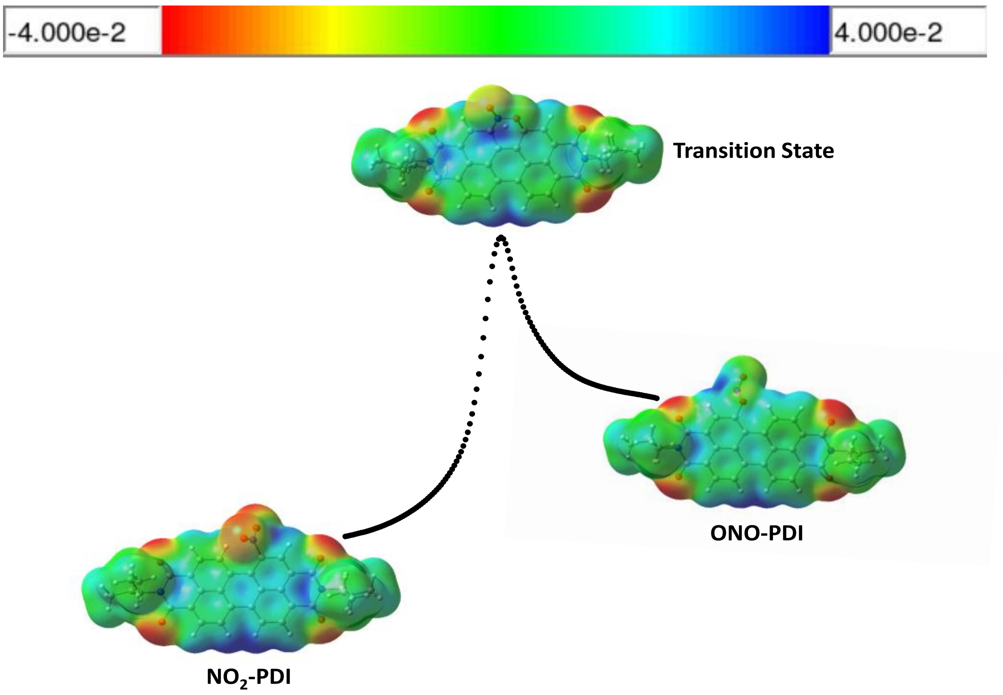

Figure S36: Molecular electrostatic potential (MESP) maps computed at the optimized geometries of NO_2-PDI, Transition state and ONO-PDI with CAM-B3LYP/6-311++G(d,p) level of theory.

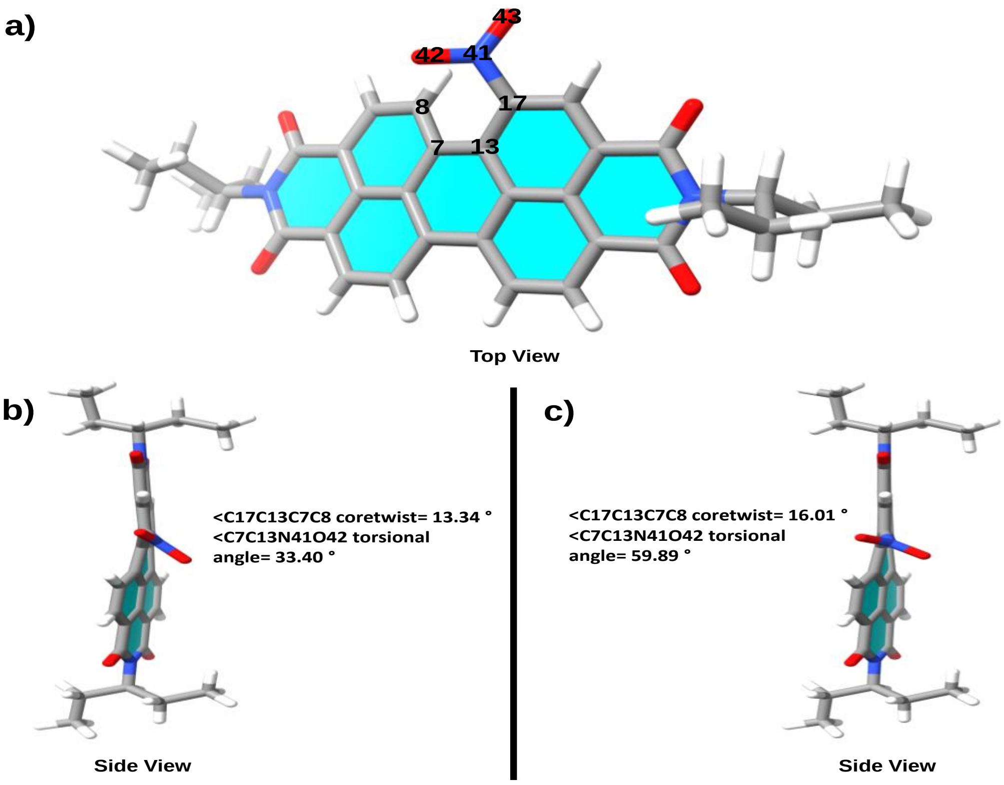

Figure S37: Conformationally relaxed singlet excited-state optimized geometry of NO_2-PDI showing a) Top view, b) Side view and c) Ground-state optimized geometry of NO_2-PDI at CAM-B3LYP/6-311++G(d,p) level of theory.

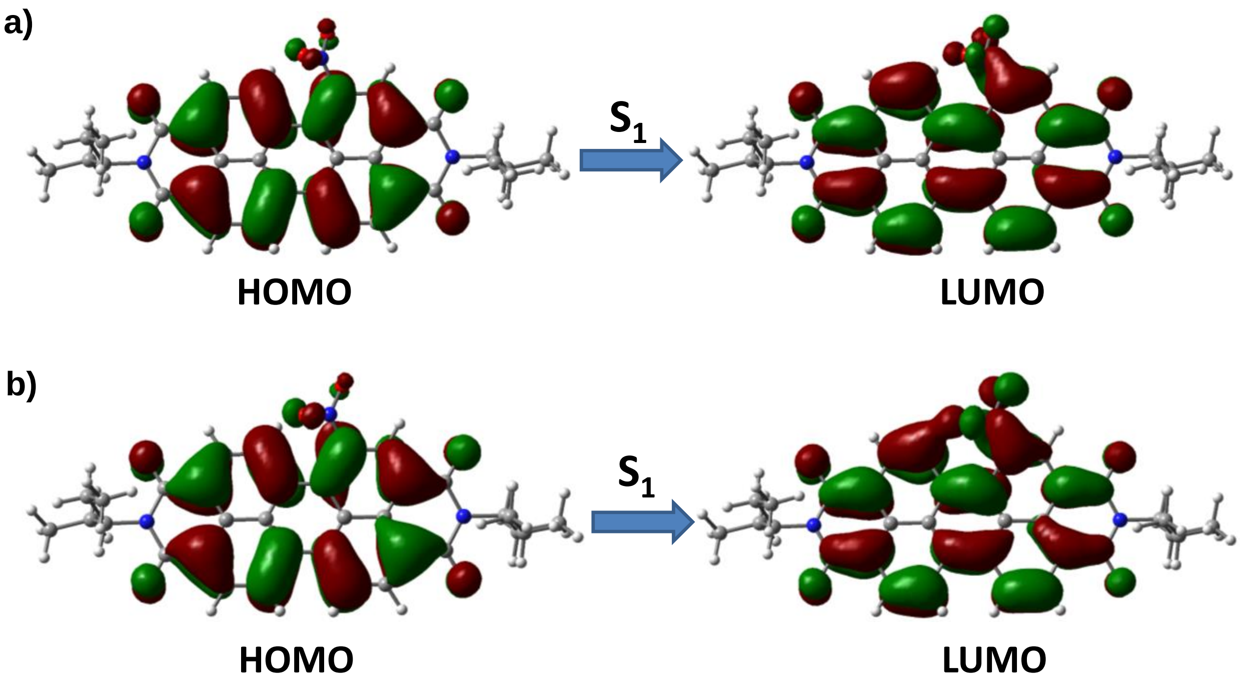

Figure S38: a) HOMO-LUMO isosurfaces (isovalue 0.02 a.u.) computed from the TD-DFT calculation with S_0 optimized geometry of NO_2-PDI and b) HOMO-LUMO isosurfaces (isovalue 0.02 a.u.) computed from the TD-DFT calculation with S_1 optimized geometry of NO_2-PDI at CAM-B3LYP/6-311++G(d,p) level of theory.

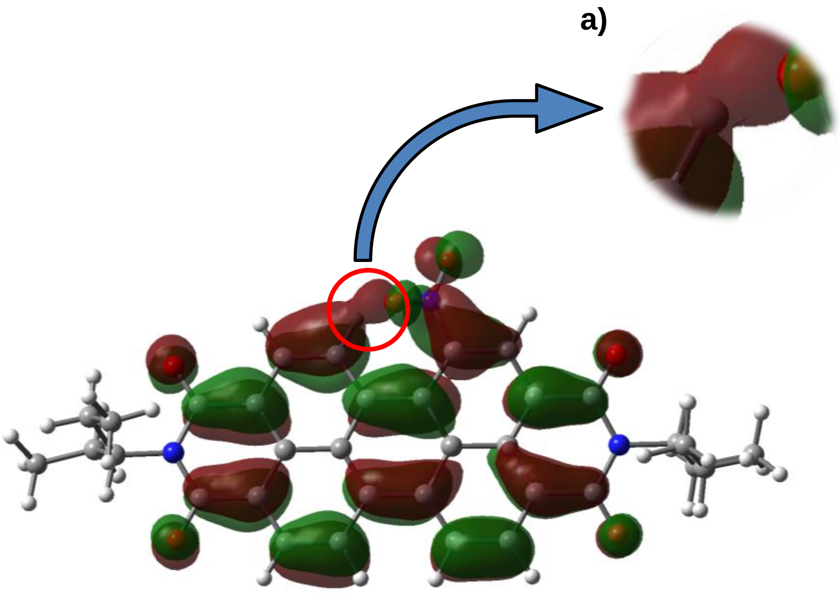

Figure S39: LUMO isosurface (isovalue 0.02 a.u.) visualized from the TD-DFT calculation with S_1 optimized geometry of NO_2-PDI. Zoomed inset a) showing the interactions between O (lone pair) and C-H orbitals in conformationally relaxed singlet excited-state of NO_2-PDI.

REFERENCES

(1) Gaussian 16, Revision C.01, Frisch, M. J.; Trucks, G. W.; Schlegel, H. B.; Scuseria, G. E.; Robb, M. A.; Cheeseman, J. R.; Scalmani, G.; Barone, V.; Petersson, G. A.; Nakatsuji, H.; Li, X.; Caricato, M.; Marenich, A. V.; Bloino, J.; Janesko, B. G.; Gomperts, R.; Mennucci, B.; Hratchian, H. P.; Ortiz, J. V.; Izmaylov, A. F.; Sonnenberg, J. L.; Williams-Young, D.; Ding, F.; Lipparini, F.; Egidi, F.; Goings, J.; Peng, B.; Petrone, A.; Henderson, T.; Ranasinghe, D.; Zakrzewski, V. G.; Gao, J.; Rega, N.; Zheng, G.; Liang, W.; Hada, M.; Ehara, M.; Toyota, K.; Fukuda, R.; Hasegawa, J.; Ishida, M.; Nakajima, T.; Honda, Y.; Kitao, O.; Nakai, H.; Vreven, T.; Throssell, K.; Montgomery, J. A., Jr.; Peralta, J. E.; Ogliaro, F.; Bearpark, M. J.; Heyd, J. J.; Brothers, E. N.; Kudin, K. N.; Staroverov, V. N.; Keith, T. A.; Kobayashi, R.; Normand, J.; Raghavachari, K.; Rendell, A. P.; Burant, J. C.; Iyengar, S. S.; Tomasi, J.; Cossi, M.; Millam, J. M.; Klene, M.; Adamo, C.; Cammi, R.; Ochterski, J. W.; Martin, R. L.; Morokuma, K.; Farkas, O.; Foresman, J. B.; Fox, D. J. Gaussian, Inc., Wallingford CT, **2016**.

(2) Lu, T.; Chen, F. Multiwfn: A Multifunctional Wavefunction Analyzer. *J. Comput. Chem.* **2012**, *33*, 580–592.

(3) Dennington, R.D.; Keith, T.A. and Millam, J.M. (**2008**) GaussView 5.0.8, Gaussian.

(4) Gao, X.; Bai, S.; Fazzi, D.; Niehaus, T.; Barbatti, M. and Thiel, W. Evaluation of Spin-Orbit Couplings with Linear-Response Time-Dependent Density Functional Methods. *J. Chem. Theory Comput*., **2017**, *13*, 515–524.

(5) Wu, Y. L.; Brown, K. E.; Wasielewski, M. R. Extending Photoinduced Charge Separation Lifetimes by Using Supramolecular Design: Guanine-Perylenediimide G-Quadruplex. *J. Am. Chem. Soc.* **2013**, *135*, 13322–13325.

(6) OriginPro, Version **2021**. OriginLab Corporation, Northampton, MA, USA.

(7) Snellenburg, J. J.; Laptenok, S.; Seger, R.; Mullen, K. M.; van Stokkum, I. H. M. Glotaran: A Java-Based Graphical User Interface for the R Package TIMP. *J. Stat. Softw.* **2012**, *49*, 1–22.

(8) Fairley, N. *Applied Surface Science Advances* (**2021**), https://doi.org/10.1016/j.apsadv.2021.100112.

(9) Dordević, L.; Milano, D.; Demitri, N.; Bonifazi, D. O-Annulation to Polycyclic Aromatic Hydrocarbons: A Tale of Optoelectronic Properties from Five- To Seven-Membered Rings. *Org. Lett.* **2020**, *22*, 4283–4288.

(10) Chen, K.-Y.; Fang, T.-C.; Chang, M.-J. *Dyes Pigm.* **2012**, *92*, 517.

(11) K. Ooms, R. E. Wasylishen, *Can. J. Chem.* **2011**, *84*, 300-308.

(12) D. Das, I. R. Laskar, A. Ghosh, A. Mondal, K. Okamoto, N. R. Chaudhuri, *J. Chem. Soc*., *Dalton Trans*. **1998**, 3987–3990.
